# Complications of Pregnancy and Birth in Women With Vascular Malformations: A Nationwide Cross‐Sectional Study

**DOI:** 10.1111/1471-0528.70205

**Published:** 2026-03-07

**Authors:** Delano J. de Oliveira Marreiros, Max M. Lokhorst, Sophie E. R. Horbach, Merel L. E. Stor, Naomi van Hout, Victor E. A. Gerdes, Wessel Ganzevoort, Chantal M. A. M. van der Horst

**Affiliations:** ^1^ Department of Plastic, Reconstructive and Hand Surgery Amsterdam University Medical Centres Amsterdam the Netherlands; ^2^ Department of Vascular Medicine Amsterdam University Medical Centres Amsterdam the Netherlands; ^3^ Department of Gynaecology and Obstetrics Amsterdam University Medical Centres Amsterdam the Netherlands

**Keywords:** complications, pregnancy, vascular malformations

## Abstract

**Objective:**

To investigate risks of pregnancy and birth in patients with peripheral vascular malformations (VMs).

**Design:**

Nationwide cross‐sectional study.

**Setting:**

Tertiary referral centre and Dutch national patient organisation.

**Population:**

Women aged ≥ 15 years diagnosed with peripheral VM of any subtype or anatomical location.

**Methods:**

Patients were invited to complete a comprehensive questionnaire about obstetric history and VM‐related symptoms during pregnancy.

**Main Outcome Measures:**

Prevalence of complications, specifically worsening of VM‐related symptoms during pregnancy, deep venous thrombosis (DVT), pulmonary embolism (PE) and postpartum haemorrhage (PPH).

**Results:**

Two‐hundred‐six women completed the questionnaire. Among 108 patients, 248 pregnancies and 204 births were reported; 98 patients were nulligravid. DVT and PE occurred in 2.5% and 0.5% of total pregnancies, respectively and only occurred in patients with extensive VMs (> 30 cm) involving at least the lower extremities (predominantly Klippel‐Trenaunay). PPH occurred in 19.6% of births, including in 63.6% of those with uterine involvement and 47.8% with genital involvement of the VM. VM‐related symptom worsening and volume increase during pregnancy occurred in 47.6% and 45.4% of patients, respectively. In multivariable regression, AVM subtype (OR 4.0, 95% CI 1.0–15.1), genital region involvement (OR 4.6, 95% CI 1.5–13.8), and a history of puberty‐related symptom progression (OR 2.6, 95% CI 1.1–6.2) were independently associated with VM‐related symptom worsening during pregnancy.

**Conclusions:**

Women with VMs appear to have a significant risk of PPH and worsening of VM‐related symptoms during pregnancy, especially if the genital region is involved. Increased thromboembolic risk appears confined to patients with extensive VMs involving the lower extremities.

## 1 Introduction

Vascular malformations (VMs) are rare congenital anomalies of the vascular system, resulting from localised errors in angiogenic development during embryogenesis [1]. VMs are categorised according to the International Society for the Study of Vascular Anomalies (ISSVA) classification and can be of venous, lymphatic, arteriovenous, capillary or combined origin [2, 3]. When localised outside the central nervous system they are referred to as peripheral VMs. The clinical presentation of peripheral VMs varies widely depending on lesion subtype, anatomical site, size and the tissues involved. Patients may present with pain, disfigurement, bleeding, functional disabilities and thrombotic complications [2, 3, 4, 5, 6, 7].

Although VMs tend to progress slowly, they can show sudden, episodic growth throughout life [8]. Trauma, infection and periods of hormonal changes, such as puberty and pregnancy, may influence VM progression through effects on vascular endothelial proliferation and blood flow, potentially exacerbating VM‐related symptoms [8, 9, 10, 11]. This suggests that reproductive hormonal changes may play an important role in VM progression, yet systematically collected data on their impact remains limited. Pregnancy poses unique challenges in women with VMs. Several case reports and case series suggest that women with large VMs, especially those located in the pelvic area and lower extremities, have a significant risk of venous thromboembolic events (VTEs) during pregnancy, severe postpartum haemorrhage (PPH) and aggravation of VM‐related symptoms during pregnancy and the early postpartum period [4, 12, 13, 14]. Additionally, a nationwide cross‐sectional study of patients with Klippel‐Trénaunay syndrome (KTS), a rare congenital disease characterised by low‐flow VMs and localised disturbed growth of bone or soft tissue, demonstrated that the risk of deep vein thrombosis (DVT) and pulmonary embolism (PE) during pregnancy was significantly higher compared to the general population [4]. However, while limited small‐scale studies are available in these high‐risk subpopulations, no studies exist assessing the risks of pregnancy and childbirth in diverse patient populations with VMs. As a result, evidence‐based guidelines for preconception counselling and obstetric management of patients with VMs are lacking, and in clinical practice some women are even advised to avoid pregnancy altogether due to the presumed risks of thrombosis, haemorrhage or symptom worsening.

To address this knowledge gap, we conducted a nationwide cross‐sectional survey study to investigate the prevalence and nature of complications during pregnancy, childbirth and the early postpartum period in women with VMs. Additionally, we examined the impact of periods of hormonal changes, such as pregnancy, puberty and hormonal treatments, on VM‐related symptoms. This study aims to improve our understanding of both obstetric risks and hormonally influenced disease progression in women with VM, to support the development of evidence‐based clinical guidelines and management strategies.

## 2 Methods

### 2.1 Study Design and Patient Selection

We conducted a cross‐sectional, questionnaire‐based study in a nationwide cohort of women with peripheral (i.e., extracranial) VMs. Women aged 15 years or older, diagnosed with any subtype of VM (e.g., venous, capillary, lymphatic, arteriovenous or combined), regardless of localisation, size or involved tissues, were eligible for inclusion. Participants were recruited from two sources: patients treated at our tertiary academic expertise centre for vascular anomalies and members of the Dutch Hemangioma and Vascular Malformations Association (HEVAS), a national patient advocacy organisation. Nearly all HEVAS participants were also treated at a university medical centre. Eligible participants received an email invitation to complete the questionnaire. This study was exempt from approval by the Amsterdam University Medical Centre’ institutional review board as it did not fall under the Dutch ‘Basic Legislation Research with Human Subjects'. All participants provided digital informed consent.

### 2.2 Questionnaire

**FIGURE 1:**
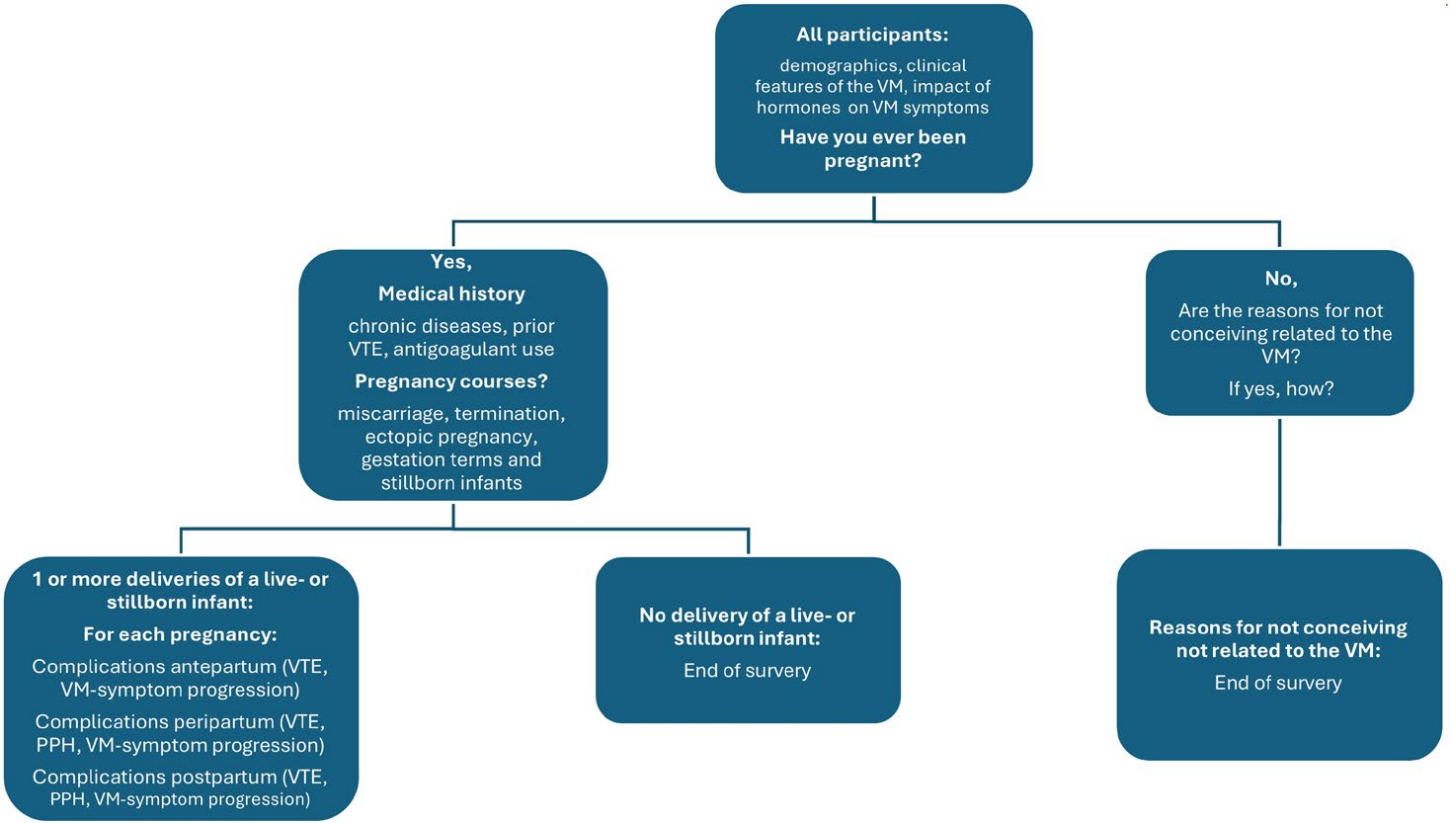
Flowchart of the structure of the online survey.

A multidisciplinary team (WG, Obstetrics and Gynaecology; VEAG, Vascular Medicine; CMAMH, SERH and DJOM, Plastic, Reconstructive and Hand Surgery) developed a comprehensive survey for this study. The questionnaire included sections on patient demographics, VM characteristics, medical history (including thromboembolic events) and obstetric history. It was adapted from a previously used questionnaire in our nationwide study on pregnancy complications in women with KTS, which was partially based on existing, validated questionnaires utilised in other patient populations [4]. These included the March of Dimes preconception and prenatal family health history questionnaire, a multi‐ethnic general health questionnaire from the HELIUS study [15] and a thrombosis screening tool developed by the Department of Vascular Medicine at Amsterdam University Medical Cent. All participants completed the introductory part of the questionnaire, with questions about clinical presentation of the VM, the impact of periods typically associated with hormonal changes (i.e., puberty, pregnancy, menstrual cycle and hormonal treatments) on VM symptoms and obstetric history. We did not directly measure hormone levels; rather, participants reported symptom changes during these presumed periods of hormonal fluctuation. Pregnancy complications were categorised into antepartum (during pregnancy), peripartum (around birth) and postpartum (until 6 weeks after birth) complications. A flowchart of the structure and content of the questionnaire is shown in Figure 1. Primary outcomes of interest were worsening of VM‐related symptoms during pregnancy, DVT, PE, and PPH. PPH was defined as an estimated blood loss > 1000 mL. To reduce the impact misclassification due to patient‐reported outcomes and recall bias, the questionnaire also captured additional objective outcomes related to haemorrhage severity, including blood transfusion, surgical haemostasis and (prolonged) postpartum hospital stay due to blood loss. Secondary outcomes were miscarriage, termination of pregnancy before 24 weeks gestation, ectopic pregnancy, intrauterine fetal death, preterm birth and caesarean section. Participants were asked directly whether these complications had occurred and could report additional complications via open‐ended responses. If a complication was reported, follow‐up questions were asked about details of treatment, hospital visits, and admissions to assess the clinical plausibility of the reported event.

For patients who were treated or gave birth at Amsterdam University Medical Centre, primary outcomes were confirmed through chart review. To ensure tailored responses, the survey used branching logic based on participants' answers. The questionnaire was developed and administered in Castor Electronic Data Capture. The original Dutch questionnaire and the English translation are provided in the Supporting Information.

### 2.3 Reference Population

To examine the relevance of our findings in comparison to the general population, we conducted a literature search for data on pregnancy outcomes and complications in the general population. We performed a comprehensive PubMed search for reference materials from population‐based cohort studies and meta‐analyses conducted within the last 15 years, focusing on complications of miscarriage, caesarean section, DVT, PE, and PPH. The search strategy is outlined in Appendix S1. We opted for the largest and most recent population‐based cohort study available to compare with the results obtained from the VM cohort. Dutch cohorts were preferred, but if not available, European or global population samples were used. For the number of PEs and DVTs extracted from general population data, only those events that occurred during pregnancy and the first 6 weeks postpartum were included to ensure consistency with our study definition of the postpartum period [16].

### 2.4 Data Analyses

For the analysis of pregnancy outcomes, all pregnancies were included. However, when assessing complications, only pregnancies that resulted in the birth of a liveborn or stillborn infant were considered, as these pregnancies encompass the full duration of gestation and the postpartum period, during which the risk of complications, such as VTE and PPH is highest. Data from current pregnancies at the time of questionnaire completion were therefore excluded from the analysis. The prevalence of complications was calculated in two ways: first, by dividing the number of patients reporting a specific complication by the total number of patients and second, by dividing the number of events by the total number of pregnancies. Subgroup analyses were performed by VM subtype and localisation.

To explore potential risk factors for VM‐related symptom worsening during pregnancy, univariable logistic regression analyses were first conducted to evaluate the association of VM subtypes, anatomical locations, VM size and a history of puberty‐related symptom progression with symptom progression during pregnancy. Variables that showed an association in the univariable analyses were considered for inclusion in the multivariable model based on their *p* values, odds ratios and clinical plausibility. Multivariable binary logistic regression was then performed to identify independent predictors of symptom worsening during pregnancy. In line with the commonly applied rule of at least 10 events per predictor variable, the number of covariates was limited to minimise overfitting. Regression analyses were not performed for PPH, DVT or PE due to insufficient event numbers for valid modelling.

To compare the prevalence of complications between the VM cohort and the general population, relative risks (RR) were calculated using the method described by Altman et al. [17] Differences in proportions were evaluated using chi‐square tests. A *p* value ≤ 0.05 was considered statistically significant.

## 3 Results

A total of 206 women completed the questionnaire, of whom 108 (52.4%) had been pregnant at least once, and 105 (51.0%) had experienced the birth of a liveborn or stillborn infant. Ninety‐eight women (47.6%) never conceived, of whom 9 (9.2%, 4.4% of the total cohort) indicated that their vascular malformation was a contributing factor in the decision to not conceive. Among those who did conceive, 27 (25.0%, 13.1% of the total cohort) reported that the presence of the vascular malformation influenced their decision on whether or not to pursue pregnancy. Patient characteristics for the full cohort, as well as for those who had a birth and those who remained nulligravid, are presented in Table 1. A comparison of patient characteristics between centre‐recruited and HEVAS‐recruited patients is provided in Appendix Table S1.

**TABLE 1:** Baseline characteristics of the study cohort.

	All patients	Patients with at least one birth	Nulligravid patients
Number of patients	206	105	98
Age in years when completing questionnaire, median (IQR)	36 (26–53)	46 (37–59)	26 (22–33)
Vascular malformations, *n* (%)			
CM	54 (26.2)	31 (29.5)	23 (23.5)
VM	130 (63.1)	68 (64.8)	61 (62.2)
AVM	28 (13.6)	13 (12.4)	14 (14.3)
LM	34 (16.5)	12 (11.4)	22 (22.4)
Unclear	25 (12.1)	15 (14.3)	9 (9.2)
Anatomical location, *n* (%)^a^			
Lower extremities	102 (49.5)	56 (53.3)	44 (44.9)
Lower extremities and trunk	28 (13.6)	15 (14.3)	12 (12.2)
Lower extremities, trunk and external genitalia	18 (8.7)	11 (10.5)	6 (6.1)
Trunk	68 (28.3)	27 (25.7)	30 (30.6)
External genitalia	37 (18.0)	23 (21.9)	13 (13.3)
Uterus	15 (7.3)	11 (10.5)	3 (3.1)
Upper extremities	51 (24.8)	24 (22.9)	25 (25.5)
Head and neck	68 (33.0)	38 (36.2)	29 (29.6)
Malformation size, *n* (%)			
< 5 cm	45 (21.8)	19 (18.1)	25 (25.5)
5–10 cm	54 (26.2)	26 (24.8)	28 (28.6)
10–20 cm	37 (18.0)	16 (15.2)	20 (20.4)
20–30 cm	10 (4.9)	4 (3.8)	5 (5.1)
> 30 cm	60 (29.1)	40 (38.1)	20 (20.4)
Tissue overgrowth, *n* (%)	98 (47.6)	55 (52.4)	42 (42.9)
Syndrome, *n* (%)	56 (27.2)	37 (35.2)	19 (19.4)
Klippel‐trenaunay	50 (24.3)	33 (31.4)	17 (17.3)
Parkes Weber	3 (1.5)	3 (2.9)	0
VM‐related symptoms, *n* (%)^b^			
Pain	153 (74.3)	78 (74.3)	73 (74.5)
Impaired mobility	88 (42.7)	39 (37.1)	48 (49.0)
Disfigurement	95 (46.1)	51 (48.6)	43 (43.9)
Bleeding	31 (15.0)	18 (17.1)	11 (11.2)
Fluid leakage	12 (5.8)	4 (3.8)	8 (8.2)
Breathing issues	13 (6.3)	7 (6.7)	6 (6.1)
Asymptomatic	11 (5.3)	4 (3.8)	7 (7.1)
Other symptoms	36 (17.5)	19 (18.1)	17 (17.3)

Abbreviations: AVM, arteriovenous malformation; CM, capillary malformation; IQR, interquartile range; LM, lymphatic malformation; VM, venous malformation.

^a^ Anatomical location categories are not mutually exclusive. For example, patients with combined lesions with involvement of the lower extremities and trunk are included under both individual locations as well as the combined category. Percentages therefore exceed 100%.

^b^ Pre‐existing VM‐related symptoms, independent of pregnancy.

### 3.1 Pregnancy Outcomes

Pregnancy outcomes are summarised in Table 2, presented both per patient and per pregnancy.

**TABLE 2:** Pregnancy outcomes of patients with vascular malformations.

Pregnancy outcomes, *n* (%)	Total patients (*n* = 108)	Total pregnancies (*n* = 248)
First trimester pregnancy loss	33 (30.6)	41 (16.4)
Miscarriage	20 (18.5)	26 (10.5)
Termination of pregnancy < 24 W	11 (10.2)	11 (4.4)
Ectopic pregnancy	4 (3.7)	4 (1.6)
Fetal death	3 (2.8)	3 (1.2)
Term birth (≥ 37 weeks)	102 (94.4)	198 (79.8)
Preterm birth (< 37 weeks)	6 (5.6)	6 (2.4)
Caesarean section	27 (25.0)	31 (12.5)

### 3.2 Venous Thromboembolic Events

Complications of pregnancy and birth are presented in Table 3. Among the 105 patients who had given birth, 4 (3.8%) experienced at least one DVT and 1 (1.0%) a PE during either pregnancy or the postpartum period. In total, 5 DVTs (2.5%) and 1 PE (0.5%) were reported across the 204 pregnancies resulting in a birth. Importantly, all DVTs and the PE occurred in patients with VMs with an associated syndrome, specifically, three with Klippel‐Trénaunay syndrome and one with Parkes Weber syndrome. All VTE patients had extensive venous malformations (> 30 cm) involving at least the lower extremities, and in two cases (50%), the genital region was also involved. No DVTs or PEs were observed in patients without an associated syndrome.

**TABLE 3:** Complications of pregnancy and birth in patients with vascular malformations.

Pregnancy‐related complications, *n* (%)	Total patients with at least one birth (*n* = 105)	Total pregnancies resulting in the birth of a live or stillborn infant (*n* = 204)
Deep vein thrombosis	4 (3.8)	5 (2.5)
During pregnancy	1 (1.0)	1 (0.5)
In postpartum period	3 (2.9)	4 (2.0)
Pulmonary embolism	1 (1.0)	1 (0.5)
During pregnancy	0	0
In postpartum period	1 (0.1)	1 (0.5)
Postpartum haemorrhage (> 1000 mL)	30 (28.6)	40 (19.6)
Blood transfusion	17 (16.2)	N/A
Hospital admission	16 (15.2)	N/A
Surgical haemostasis	10 (9.5)	N/A
Aggravated VM symptoms	50 (47.6)	N/A
Pain	36 (34.3)	N/A
Impaired mobilty	23 (21.9)	N/A
Disfigurement	17 (16.2)	N/A
Bleeding	8 (7.6)	N/A
Increase in VM volume	49 (45.4)	N/A

Abbreviations: N/A, not available; VM, vascular malformation.

### 3.3 Postpartum Haemorrhage

Among the 105 patients who had given birth, 30 (28.6%) experienced at least one episode of PPH. This corresponded to 40 PPH events (19.6%) across 204 births. PPH occurred in 7 of 11 (63.6%) patients with uterine involvement and in 11 of 23 (47.8%) patients with VMs involving the external genitalia. In the subgroup of patients without uterine or genital VM involvement, 16 of 81 (19.7%) experienced PPH.

### 3.4 VM‐Related Symptom Worsening and Volume Increase During Pregnancy

Aggravation of VM‐related symptoms during pregnancy was reported by 50 (47.6%) patients. The most commonly reported symptom was increased pain (34.3%), followed by impaired mobility (21.9%). Forty‐nine (45.4%) patients reported an increase in VM volume during pregnancy. In the multivariable logistic regression analysis, genital region involvement (OR 4.6, 95% CI 1.5–13.8, *p* = 0.007), the presence of an arteriovenous malformation (AVM) (OR 4.0, 95% CI 1.0–15.1, *p* = 0.043) and a history of symptom worsening during puberty (OR 2.6, 95% CI 1.1–6.2, *p* = 0.032) were independently associated with symptom worsening during pregnancy. VM size ≥ 30 cm was not associated with symptom worsening (OR 1.65, 95% CI 0.7–4.0, *p* = 0.273).

### 3.5 Impact of Hormonal Changes on VM‐Related Symptoms

VM‐related symptom worsening was most frequently reported during puberty (49.0%), followed by pregnancy (47.6%), the menstrual cycle (29.1%) and hormonal contraceptive use (19.9%), as listed in Table 4. Among the 50 patients who reported symptom worsening during pregnancy, 26 (52.0%) patients had also experienced symptom aggravation during puberty and in 21 (42.0%) patients, symptoms were also related to the menstrual cycle.

**TABLE 4:** Patient‐reported impact of factors related with hormonal changes on vascular malformation symptoms.

Hormonal factor	N reporting exposure	N reporting change in symptoms, *n* (%)	Symptom worsening, *n* (%)	Improvement, *n* (%)	No change, *n* (%)
Puberty	206	102 (49.5)	101 (49.0)	1 (0.5)	104 (50.5)
Menstrual cycle	206	60 (29.1)	N/A	N/A	146 (70.9)
Hormonal contraceptives	171	45 (26.3)	34 (19.9)	11 (6.4)	126 (73.7)
Pregnancy	105	51 (48.6)	50 (47.6)	1 (1.0)	54 (51.4)

### 3.6 Comparison to Reference Population

In comparison to the general population, patients with VMs had significantly higher rates of DVT (RR 26.9, 95% CI 11.3–64.0, *p* < 0.0001), PE (RR 11.8, 95% CI 1.7–83.4, *p* = 0.0134), and PPH (RR 3.7, 95% CI 2.8–4.9, *p* < 0.0001) (Table 5). In contrast, the risk of miscarriage was lower in the VM cohort (RR 0.69, 95% CI 0.48–0.99, *p* = 0.0416), and caesarean section rates were comparable between both groups (RR 0.9, 95% CI 0.6–1.1, *p* = 0.1873).

**TABLE 5:** Comparison of pregnancy complications in the vascular malformation cohort with data from large population‐based cohorts from the literature.

Complication	Vascular malformation cohort, %	General population‐based cohorts, %	Relative risk (95% CI)	*p*
Deep vein thrombosis	2.5	0.09 [16]	26.9 (11.3–64.0)	< 0.0001
Pulmonary embolism	0.5	0.05 [16]	11.8 (1.7–83.4)	0.0134
Postpartum haemorrhage	19.6	5.3 [18]	3.7 (2.8–4.9)	< 0.0001
Miscarriage	10.5	15.3 [19]	0.69 (0.48–0.99)	0.0416
Caesarean section	12.5	15.6 [20]	0.9 (0.6–1.1)	0.1873

*Note:* Complications are presented as a percentage of the total number of pregnancies for both the VM cohort, as well as the general population‐based cohorts.

## 4 Discussion

### 4.1 Main Findings

In this study, we evaluated complications of pregnancy and birth across a broad population of women with diverse VM subtypes and anatomical locations. Our results indicate that, although the overall incidence of DVT and PE during pregnancy or in the postpartum period was significantly higher in women with VMs compared to the general population, all VTEs were observed in patients with extensive VMs involving at least the lower extremities, all of whom had an associated syndrome, predominantly KTS. No VTEs were reported in the rest of the study population. The risk of PPH was significantly elevated, particularly in women with a VM involving the genital tract. Additionally, approximately half of the participants reported an increase in VM volume and worsening of VM‐related symptoms during pregnancy, predominantly manifesting as increased pain and reduced mobility. The presence of an AVM, involvement of the genital region and a history of symptom aggravation during puberty appeared to be independent risk factors for worsening of VM‐related symptoms during pregnancy.

### 4.2 Interpretation

The exclusive occurrence of pregnancy‐ and postpartum‐related DVT and PE in patients with an associated syndrome in our study aligns with the findings of a nationwide cohort study by Horbach et al., who reported a significantly increased thromboembolic risk during pregnancy and the postpartum period in patients with KTS [4]. Similarly, a study Marvin et al. found that 20% of patients with KTS experienced a VTE at some point in their lifetime, confirming a markedly increased baseline thrombotic risk in this population [21]. However, while they concluded that the lifetime risk of VTEs was elevated, this risk was independent of pregnancy status. Nevertheless, among KTS patients who had been pregnant, 39% of all VTEs reported in their study occurred during pregnancy or the postpartum period, indicating that pregnancy remains a period of increased risk within an already thrombosis‐prone group. In our cohort, all patients who developed VTEs not only had an associated syndrome but also extensive VMs involving at least the lower extremities, with genital involvement in half the cases. It therefore remains uncertain whether the observed increase in thromboembolic risk is primarily driven by the syndromal context, or by lesion‐related characteristics such as size and location. Nevertheless, these findings suggest that elevated thrombotic risk is not uniformly present across the broader VM population, but may be specific to extensive malformations, as seen in syndromal conditions such as KTS. Individualised risk assessment remains essential, and prophylactic anticoagulation should be considered in selected high‐risk cases, particularly in patients with extensive VMs involving the lower extremities and when the genital region is affected.

PPH was significantly more common in women with VMs in our cohort compared to the general population. This is consistent with the findings by Horbach et al., who reported that severe PPH occurred in 11% of pregnancies in women with KTS compared to 5.8% in the general population. The elevated risk in our study was especially pronounced among participants with VMs involving the uterus or genital tract, where the presence of abnormal vasculature may directly impair uterine contractility or increase susceptibility to trauma and bleeding during both vaginal birth and a caesarean section. While our overall cohort included women with VMs in other anatomical sites, the increased incidence of PPH might be mostly driven by those with uterine or genital lesions. Uterine AVMs, though rare, are well documented causes of primary and delayed PPH, frequently necessitating uterine artery embolization or hysterectomy [22, 23, 24]. These observations underscore the importance of targeted obstetric planning and interdisciplinary management in patients with reproductive tract‐involved VMs, including pre‐pregnancy imaging and delivery in a vascular anomalies expertise centre. Further research is needed to better define which lesion‐specific features, such as size, flow dynamics or depth of tissue involvement, confer the greatest haemorrhagic risk and to determine which delivery strategies may help mitigate the risk of PPH in this population.

Our study found that approximately half of the women with vascular malformations experienced an increase in VM volume and worsening of VM‐related symptoms during pregnancy, most commonly presenting as pain and impaired mobility. These findings are in line with earlier clinical observations by Horbach et al., who reported symptom aggravation in approximately half of the pregnant women with KTS [4]. Similarly, findings by Dubois demonstrated that 91% of KTS patients experienced important volume increase of their VMs during pregnancy. The exacerbation of symptoms is likely multifactorial, driven by pregnancy‐related increases in circulating blood volume, venous pressure, and hormonal fluctuations.

Mounting immunohistochemical evidence also supports a hormonal influence on vascular malformation behaviour [8, 10, 25, 26]. Notably, Utami et al. demonstrated the expression of oestrogen, progesterone, growth hormone and follicle‐stimulating hormone receptors in vasoproliferative areas of congenital AVMs, which could explain sudden episodic growth of VMs during both puberty and pregnancy [10]. Furthermore, Kulungowski et al. showed that growth hormone receptors are overexpressed and principally located in the vessels of several VM subtypes [25]. These findings suggest that certain VMs may be hormonally responsive and thus subject to progression under the influence of endocrine fluctuations. Consistent with this, VM‐related symptom progression during pregnancy in our cohort was significantly more frequently observed in patients who had also experienced symptom worsening during puberty, further supporting the role of hormonal sensitivity in a subset of individuals. Clinically, this underscores the importance of preconceptional counselling for patients with hormonally responsive VMs. In addition, several participants in our study reported symptom changes in response to hormonal contraceptive use and fluctuations of VM symptoms during the menstrual cycle. While data are currently limited, these observations highlight a need for individualised decision‐making when prescribing hormonal medication in this population. A better understanding of hormone‐receptor expression patterns in different VM subtypes may eventually support more personalised approaches to both preconception risk assessment and symptom management and could potentially guide the development of hormone‐targeted therapeutic strategies in selected patients.

### 4.3 Strengths and Limitations

This is the largest study to date examining pregnancy‐related complications and hormonal change‐associated symptom progression in women with VMs. Unlike previous studies, mostly limited to case reports or small cohorts of syndromal patients, our diverse cohort, encompassing patients with various VM subtypes and anatomical locations, enhances generalizability to routine clinical care. The questionnaire, developed by a multidisciplinary team using branching logic, enabled tailored and detailed data collection. Furthermore, recruitment via a tertiary care centre and a national patient advocacy group likely enriched the number of individuals with strong diagnostic awareness and health literacy, thereby improving the reliability of patient‐reported outcomes. However, recruitment from two different sources could have also introduced bias, as both disease severity and care among patients recruited through the HEVAS might have differed from those receiving care at an expertise centre. However, as nearly all patients recruited through the national patient advocacy organisation were also under the care of a university medical centre, these groups were unlikely to differ substantially in terms of patient population or type or quality of care received. Nevertheless, this recruitment strategy may have led to selection bias, as it may have resulted in a higher proportion of women with more severe VMs, potentially leading to overestimation of complication rates and limiting generalizability to those with milder forms. Another limitation of this study is its cross‐sectional design, which is inherently subject to recall bias, particularly in relation to past obstetric events and the timing or severity of VM‐related symptoms. Participants may have misremembered or underreported complications, especially for pregnancies that occurred many years ago. Furthermore, the study relied predominantly on self‐reported data rather than clinical records, which may have affected the accuracy of reported outcomes such as miscarriage, PPH, DVT or PE. Although the questionnaire included follow‐up items to clarify and contextualise complications, we were only able to verify complications for patients treated at our centre through chart review, but this was not feasible for the rest of the respondents. For PPH specifically, we performed a sensitivity analysis using blood transfusion, (prolonged) hospital admission due to blood loss, and surgical haemostasis as proxy indicators of severe haemorrhage. However, these data were available at the patient level only and not per individual pregnancy. Furthermore, although participants were asked to indicate during which pregnancy complications occurred, multiple pregnancies within the same woman are inherently correlated rather than independent observations. Prior complications may influence both the risk of subsequent complications and future reproductive choices, which could affect per‐pregnancy risk estimates and should be considered when interpreting these results. Lastly, we did not collect several key covariates that are known to influence pregnancy outcomes, including maternal age at the time of pregnancy, body mass index, smoking status or family history of thrombosis. This limitation partly reflects the need to balance comprehensiveness with respondent burden and minimise questionnaire length. Despite being the largest study in this field, the number of respondents and pregnancies remains modest. Larger, preferably international registries are needed to validate our findings, explore the influence of interventions on outcomes, and better define high‐risk subgroups to guide evidence‐based recommendations.

## 5 Conclusion

This nationwide study provides novel insights into pregnancy‐related risks and symptom progression in women with VMs. PPH was significantly more common than in the general population, particularly in patients with genital or uterine involvement. Nearly half of the participants reported symptom worsening during pregnancy, which appeared more common in those with AVMs, genital region involvement, or a history of puberty‐related symptom progression. In contrast, the risk of VTEs did not appear to be generally elevated across all patients with VMs, as events were confined to patients with extensive VMs involving the lower extremities and genital region. These findings highlight the need for personalised reproductive counselling, multidisciplinary pregnancy care, and targeted risk assessment in women with VMs considering pregnancy. Larger registration and prospective studies are warranted to confirm these observations in more detail (including interventions) and further support the development of evidence‐based clinical guidelines.

## Author Contributions

**Delano J. de Oliveira Marreiros:** conceptualization; data curation; formal analysis; methodology; writing – original draft. **Max M. Lokhorst:** conceptualization; data curation; methodology; writing – review and editing. **Sophie E. R. Horbach:** conceptualization; supervision; methodology; writing – review and editing. **Merel L. E. Stor:** supervision; writing – review and editing. **Naomi van Hout:** supervision; writing – review and editing. **Victor E. A. Gerdes:** supervision; methodology; writing – review and editing. **Wessel Ganzevoort:** conceptualization; supervision; methodology; writing – review and editing. **Chantal M. A. M. van der Horst:** conceptualization; supervision; writing – review and editing.

## Funding

This study was funded by a grant from the Dutch Hemangioma and Vascular Malformations Association (HEVAS), a national patient advocacy organisation. HEVAS assisted in distributing the questionnaire among its members, who are patients with vascular malformations. The organisation was not otherwise involved in the design, conduct or writing of the study.

## Ethics Statement

This study was exempt from approval by the Amsterdam University Medical Centre’ institutional review board as it did not fall under the Dutch ‘Basic Legislation Research with Human Subjects'. All participants provided digital informed consent.

## Conflicts of Interest

The authors declare no conflicts of interest.

## Supporting information


**Data S1:** bjo70205‐sup‐0001‐Supinfo1.docx.

**Data S2:** bjo70205‐sup‐0002‐Supinfo2.docx.

**Table S1:** Baseline characteristics of patients who had at least one birth, comparing patients recruited through Amsterdam University Medical Centre and HEVAS.

## Data Availability

The data that support the findings of this study are available on request from the corresponding author. The data are not publicly available due to privacy or ethical restrictions.

## References

[1] L. B. Meijer‐Jorna , C. M. van der Loos , O. J. de Boer , C. M. van der Horst , and A. C. van der Wal , “Microvascular Proliferation in Congenital Vascular Malformations of Skin and Soft Tissue,” Journal of Clinical Pathology 60, no. 7 (2007): 798–803. 16816171 10.1136/jcp.2006.038885 PMC1995770

[2] R. Dasgupta and S. J. Fishman , “ISSVA Classification,” Seminars in Pediatric Surgery 23, no. 4 (2014): 158–161. 25241091 10.1053/j.sempedsurg.2014.06.016

[3] M. Wassef , F. Blei , D. Adams , et al., “Vascular Anomalies Classification: Recommendations From the International Society for the Study of Vascular Anomalies,” Pediatrics 136, no. 1 (2015): e203–e214. 26055853 10.1542/peds.2014-3673

[4] S. E. Horbach , M. M. Lokhorst , C. E. Oduber , S. Middeldorp , J. A. van der Post , and C. M. van der Horst , “Complications of Pregnancy and Labour in Women With Klippel‐Trénaunay Syndrome: A Nationwide Cross‐Sectional Study,” BJOG: An International Journal of Obstetrics Gynaecology 124, no. 11 (2017): 1780–1788. 28432715 10.1111/1471-0528.14698

[5] S. E. Horbach , M. M. Lokhorst , P. Saeed , C. M. de Goüyon Matignon Pontourau , A. Rothová , and C. M. van der Horst , “Sclerotherapy for Low‐Flow Vascular Malformations of the Head and Neck: A Systematic Review of Sclerosing Agents,” Journal of Plastic, Reconstructive & Aesthetic Surgery 69, no. 3 (2016): 295–304. 10.1016/j.bjps.2015.10.045 26723834

[6] S. E. R. Horbach , C. van der Horst , F. Blei , et al., “Development of an International Core Outcome Set for Peripheral Vascular Malformations: The OVAMA Project,” British Journal of Dermatology 178, no. 2 (2018): 473–481. 28986976 10.1111/bjd.16029

[7] D. J. de Oliveira Marreiros , M. M. Lokhorst , D. A. Young‐Afat , et al., “Measuring Health‐Related Quality of Life in Patients With Peripheral Vascular Malformations With PROMIS Computerized Adaptive Tests,” Journal of Plastic, Reconstructive & Aesthetic Surgery 106 (2025): 401–408. 10.1016/j.bjps.2025.05.036 40482632

[8] L. J. Duyka , C. Y. Fan , J. M. Coviello‐Malle , L. Buckmiller , and J. Y. Suen , “Progesterone Receptors Identified in Vascular Malformations of the Head and Neck,” Otolaryngology and Head and Neck Surgery 141, no. 4 (2009): 491–495. 10.1016/j.otohns.2009.06.012 19786218

[9] M. P. Kohout , M. Hansen , J. J. Pribaz , and J. B. Mulliken , “Arteriovenous Malformations of the Head and Neck: Natural History and Management,” Plastic and Reconstructive Surgery 102, no. 3 (1998): 643–654. 9727427 10.1097/00006534-199809030-00006

[10] A. M. Utami , J. B. G. Halfwerk , O. J. de Boer , et al., “Relative Expression of Hormone Receptors by Endothelial and Smooth Muscle Cells in Proliferative and Non‐Proliferative Areas of Congenital Arteriovenous Malformations,” European Journal of Medical Research 28, no. 1 (2023): 449. 37864259 10.1186/s40001-023-01436-5 PMC10588228

[11] S. Delplanque , M. Le Lous , M. Proisy , et al., “Fertility, Pregnancy, and Clinical Outcomes After Uterine Arteriovenous Malformation Management,” Journal of Minimally Invasive Gynecology 26, no. 1 (2019): 153–161. 29772406 10.1016/j.jmig.2018.05.001

[12] A. Keepanasseril , K. Keerthana , A. Keepanasseril , D. K. Maurya , D. Kadambari , and S. Sistla , “Pregnancy in Women With Klippel‐Trenaunay Syndrome: Report of Three Pregnancies in a Single Patient and Review of Literature,” Obstetric Medicine 10, no. 4 (2017): 177–182. 29225678 10.1177/1753495X17719181 PMC5714109

[13] T. Güngor Gündoğan and Y. Jacquemyn , “Klippel‐Trenaunay Syndrome and Pregnancy,” Obstetrics and Gynecology International 2010 (2010): 706850. 21209709 10.1155/2010/706850 PMC3010666

[14] S. M. Kim , W. K. Jang , J. C. Park , J. H. Rhee , J. I. Kim , and J. G. Bae , “Arteriovenous Malformation in Uterine Cervix During Pregnancy,” Obstetrics & Gynecology Science 57, no. 2 (2014): 155–159. 24678490 10.5468/ogs.2014.57.2.155 PMC3965700

[15] K. Stronks , M. B. Snijder , R. J. Peters , M. Prins , A. H. Schene , and A. H. Zwinderman , “Unravelling the Impact of Ethnicity on Health in Europe: The HELIUS Study,” BMC Public Health 13 (2013): 402. 23621920 10.1186/1471-2458-13-402 PMC3646682

[16] J. Philipson , E. Thunström , T. Svanvik , et al., “Incidence and Time Trends of Pregnancy‐Related First‐Time Venous Thromboembolism: A 33‐Year Swedish Birth Registry Study,” Journal of Thrombosis and Haemostasis 23 (2025): 2473–2482. 40234144 10.1016/j.jtha.2025.03.009

[17] D. G. Altman , Practical Statistics for Medical Research (Chapman and Hall/CRC, 1990).

[18] G. van Stralen , J. F. von Schmidt Auf Altenstadt , K. W. Bloemenkamp , J. van Roosmalen , and C. W. Hukkelhoven , “Increasing Incidence of Postpartum Hemorrhage: The Dutch Piece of the Puzzle,” Acta Obstetricia et Gynecologica Scandinavica 95, no. 10 (2016): 1104–1110. 27460955 10.1111/aogs.12950

[19] S. Quenby , I. D. Gallos , R. K. Dhillon‐Smith , et al., “Miscarriage Matters: The Epidemiological, Physical, Psychological, and Economic Costs of Early Pregnancy Loss,” Lancet 397, no. 10285 (2021): 1658–1667. 33915094 10.1016/S0140-6736(21)00682-6

[20] J. Zhang , C. Geerts , C. Hukkelhoven , P. Offerhaus , J. Zwart , and A. de Jonge , “Caesarean Section Rates in Subgroups of Women and Perinatal Outcomes,” BJOG: An International Journal of Obstetrics and Gynaecology 123, no. 5 (2016): 754–761. 26216434 10.1111/1471-0528.13520

[21] E. K. Marvin , J. J. Schoch , H. Nguyen , et al., “Venous Thromboembolic and Bleeding Complications Among Pregnant Women With Klippel‐Trenaunay Syndrome,” Journal of the American Academy of Dermatology 81, no. 6 (2019): 1277–1282. 30991120 10.1016/j.jaad.2019.04.018

[22] D. J. Yoon , M. Jones , J. A. Taani , C. Buhimschi , and J. D. Dowell , “A Systematic Review of Acquired Uterine Arteriovenous Malformations: Pathophysiology, Diagnosis, and Transcatheter Treatment,” American Journal of Perinatology Reports 6, no. 1 (2016): e6–e14. 26929872 10.1055/s-0035-1563721 PMC4737639

[23] N. Gallagher , M. Cincotta , H. Keblawi , D. Jude , and M. Korona , “Uterine Arteriovenous Malformation Leading to Postpartum Hemorrhage: A Case Report,” Case Reports in Women's Health 28 (2020): e00260. 10.1016/j.crwh.2020.e00260 PMC7559227 33088725

[24] S. M. Kelly , A. M. Belli , and S. Campbell , “Arteriovenous Malformation of the Uterus Associated With Secondary Postpartum Hemorrhage,” Ultrasound in Obstetrics & Gynecology 21, no. 6 (2003): 602–605. 12808679 10.1002/uog.148

[25] A. M. Kulungowski , A. H. Hassanein , V. Nosé , et al., “Expression of Androgen, Estrogen, Progesterone, and Growth Hormone Receptors in Vascular Malformations,” Plastic and Reconstructive Surgery 129, no. 6 (2012): 919e–924e. 10.1097/PRS.0b013e31824ec3fb 22634690

[26] A. M. Utami , S. E. R. Horbach , L. B. Meijer‐Jorna , et al., “Microvascular Proliferation in Arteriovenous Malformation of the Hand Worsens During Pregnancy: A Case Report,” Annals of Medicine and Surgery 85, no. 4 (2023): 1262–1269. 37113922 10.1097/MS9.0000000000000507 PMC10129217

