# Supplementary material for: Complications of Pregnancy and Birth in Women With Vascular Malformations: A Nationwide Cross‐Sectional Study
Source: BJOG. 2026 Mar 7;133(7):1493–501. doi: 10.1111/1471-0528.70205 (PMC13143545; doi:10.1111/1471-0528.70205)
Supplement: Supplementary file 1 — Data S1: bjo70205‐sup‐0001‐Supinfo1.docx. [file BJO-133-1493-s002.docx]

Survey Package 'Onderzoek vasculaire malformaties en zwangerschap'

## Onderzoek vasculaire malformaties en zwangerschap - Introduction

Op de afdeling Plastische chirurgie in het Amsterdam Universitair Medisch Centrum (AUMC) zijn wij bezig met een nieuw onderzoek naar vasculaire malformaties (aangeboren vaatafwijkingen). Op dit moment is er weinig duidelijkheid over hoe vrouwelijke patiënten met vasculaire malformaties moeten worden geadviseerd en behandeld wanneer zij zwanger willen worden of zwanger zijn.

Om deze reden onderzoeken wij in de gehele Nederlandse vrouwelijke patiëntengroep of er problemen zijn geweest met zwanger worden en of er problemen waren tijdens of in de periode kort na de zwangerschap. Hierdoor kunnen wij in de toekomst patiënten met vasculaire malformaties beter adviseren, en eventueel beter begeleiden rondom de zwangerschap.

Hier volgt nu de eenmalige vragenlijst. De duur van het invullen van de vragenlijst is afhankelijk van het aantal zwangerschappen dat u heeft gehad. Dit varieert van enkele minuten (geen zwangerschap) tot maximaal 20 minuten. Ook als u nog nooit zwanger bent geweest willen wij u vragen deze vragenlijst in te vullen, zodat wij een zo compleet mogelijk beeld krijgen van de vrouwelijke patiëntengroep met vasculaire malformaties. **Als u de vragenlijst tussentijds sluit of wegklikt, kunt u deze later niet opnieuw openen en verdergaan waar u gebleven was. Zorg er daarom voor dat u voldoende tijd heeft om de vragenlijst in één**

### keer volledig in te vullen.

Uw medewerking zal ons verder helpen in een beter begrip van de aandoening en betere begeleiding en behandeling voor vrouwen met vasculaire malformaties.

Survey 'Vasculaire malformaties en zwangerschap vragenlijst'

# Vasculaire malformaties en zwangerschap vragenlijst - Persoonsgegevens

### Number Question Answers

Deze vragenlijst is bedoeld voor vrouwen van 15 jaar en ouder met een aangeboren vaatafwijking (vasculaire malformatie). Als u hier niet onder valt, hoeft u de vragenlijst niet in te vullen.

- 1. Via welke route heeft u de uitnodiging ontvangen om aan dit onderzoek deel te nemen? (Let op: als u zowel via HEVAS als via het Amsterdam UMC bent benaderd, hoeft u de vragenlijst maar één keer in te vullen.)


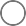
 E-mail van het Amsterdam UMC
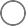
 E-mail van de HEVAS


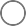
 Besloten facebookgroep

- 1. Verleent u toestemming voor het gebruik van uw persoonsgegevens voor deze wetenschappelijke studie? De gegevens worden anoniem in de studie verwerkt.


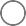
 Ja
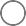
 Nee

Uw gegevens worden anoniem verwerkt.

#### 1.2.2 If 'Verleent u toestemming voor het gebruik van uw persoonsgegevens voor deze wetenschappelijke studie? De gegevens worden anoniem in de studie verwerkt.' is equal to 'Ja' answer this question:

Geboortedatum

*(dd-mm-yyyy)*

- 1. Wat is uw huidige leeftijd? Jaren

#### If 'Verleent u toestemming voor het gebruik van uw persoonsgegevens voor deze wetenschappelijke studie? De gegevens worden anoniem in de studie verwerkt.' is equal to 'Ja' answer this question:

Naam (voor- en achternaam)

#### If 'Verleent u toestemming voor het gebruik van uw persoonsgegevens voor deze wetenschappelijke studie? De gegevens worden anoniem in de studie verwerkt.' is equal to 'Ja' answer this question:

Verleent u toestemming voor het opvragen van medische gegevens bij uw behandelaar in het kader van wetenschappelijk onderzoek naar vasculaire malformaties? Deze gegevens zullen anoniem worden verwerkt.


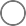
 Ja
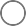
 Nee

# Vasculaire malformaties en zwangerschap vragenlijst - Type vasculaire malformatie (aangeboren vaatafwijking)

### Number Question Answers

Hierna volgen een aantal vragen over het type vasculaire malformatie en de klachten die u hiervan ervaart.

- 1. In welk ziekenhuis bent u bekend met de vasculaire malformatie (aangeboren vaatafwijking)?
  2. Welk type vasculaire malformatie (aangeboren vaatafwijking) heeft u? Als u een combinatie heeft kunt u meerdere types aanvinken.


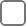
 Capillaire malformatie (wijnvlek)


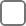
 Veneuze malformatie (spataders/aangeboren afwijking van de aders)


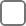
 Lymfatische malformatie (afwijking van de lymfevaten)


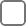
 Arterioveneuze malformatie (afwijking van de slagaders)


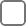
 Onduidelijk

- 1. Op welke plek(ken) zit de vasculaire malformatie?
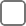
 Hoofd/hals


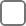
 Borst/buik/bekken
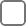
 Armen/handen


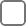
 Benen/voeten
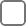
 Genitaal regio
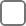
 Baarmoeder

- 1. Hoe groot is de vasculaire malformatie? (Meet of maak een inschatting van de maximale diameter die aan de buitenkant zichtbaar is.)


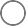
 Kleiner dan 5 cm
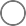
 5-10 cm


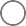
 10-20 cm


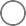
 20-30 cm


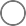
 Groter dan 30 cm

- 1. Heeft u lichaamsdelen die afwijkend zijn in grootte/omvang? Hiertoe behoort bijvoorbeeld ook beenlengteverschil.


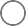
 Ja
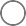
 Nee

#### If 'Heeft u lichaamsdelen die afwijkend zijn in grootte/omvang? Hiertoe behoort bijvoorbeeld ook

***beenlengteverschil.' is equal to 'Ja' answer this question:***

Welke lichaamsdelen zijn afwijkend in grootte/omvang? Meerdere antwoorden mogelijk. Vink alle toepasselijke opties aan.


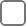
 Hoofd/hals
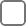
 Armen


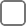
 Handen/vingers
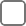
 Benen


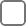
 Voeten/tenen


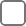
 Borst/buik/bekken/rug
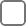
 Genitaal regio

#### If 'Heeft u lichaamsdelen die afwijkend zijn in grootte/omvang? Hiertoe behoort bijvoorbeeld ook

***beenlengteverschil.' is equal to 'Ja' answer this question:***

Hoe verschilt dit lichaamsdeel in grootte/omvang?


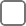
 Langer
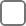
 Dikker
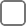
 Korter
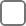
 Dunner
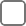
 Anders

#### If 'Heeft u lichaamsdelen die afwijkend zijn in grootte/omvang? Hiertoe behoort bijvoorbeeld ook

***beenlengteverschil.' is equal to 'Ja' answer this question:***

Indien nodig toelichting:

- 1. Heeft u de vasculaire malformatie(s) als onderdeel van een syndroom? Bijvoorbeeld het Klippel-Trenaunay syndroom.


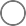
 Ja
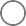
 Nee

#### If 'Heeft u de vasculaire malformatie(s) als onderdeel van een syndroom? Bijvoorbeeld het Klippel-Trenaunay

***syndroom.' is equal to 'Ja' answer this question:***

Welk syndroom heeft u?

# Vasculaire malformaties en zwangerschap vragenlijst - Symptomen van de vasculaire malformatie en invloed van hormonen

### Number Question Answers

De volgende vragen over de symptomen die u ervaart ten gevolge van de vasculaire malformatie en welke factoren hier invloed op hebben gehad.

- 1. Welke symptomen ervaart u aan de vasculaire malformatie? Meerdere antwoorden mogelijk. Vink alle toepasselijke opties aan.


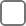
 Pijn


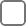
 Bloedingen


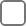
 Bewegingsbeperking


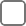
 Klachten van verstoord uiterlijk
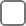
 Ademhalingsproblemen


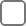
 Lekkage van vocht
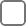
 Geen klachten


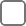
 Anders

- 1. Indien nodig toelichting
  2. Zijn de klachten van de vasculaire malformatie begonnen rond de puberteit?


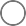
 Ja
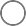
 Nee

- 1. Zijn de klachten van de vasculaire malformatie veranderd rond de puberteit?


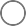
 Ja
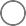
 Nee

- 1. Indien ja op voorgaande vragen: hoe zijn de klachten van de vasculaire malformatie begonnen of veranderd in de puberteit?
  2. Zijn de klachten/symptomen die u ervaart ten gevolge van uw vasculaire malformatie gebonden aan uw menstruele cyclus?


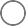
 Ja
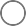
 Nee

- - 1. ***If 'Zijn de klachten/symptomen die u ervaart ten gevolge van uw vasculaire malformatie gebonden aan uw menstruele cyclus?' is equal to 'Ja' answer this question:*** Hoe veranderen de klachten/symptomen tijdens uw cyclus?
  1. Heeft u ooit hormonale anticonceptie gebruikt? Bijvoorbeeld de anticonceptiepil of een hormoonspiraal.


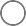
 Ja
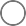
 Nee

#### If 'Heeft u ooit hormonale anticonceptie gebruikt? Bijvoorbeeld de anticonceptiepil of een hormoonspiraal.' is equal to 'Ja' answer this question:

Zijn de klachten van de vasculaire malformatie veranderd tijdens het gebruik van de hormonale anticonceptie?


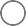
 Ja
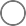
 Nee

#### If 'Zijn de klachten van de vasculaire malformatie veranderd tijdens het gebruik van de hormonale

***anticonceptie?' is equal to 'Ja' answer this question:*** Wat veranderde er aan de klachten tijdens het gebruik van hormonale anticonceptie?

# Vasculaire malformaties en zwangerschap vragenlijst - Verloskundige voorgeschiedenis

### Number Question Answers

De volgende vragen gaan over uw verloskundige voorgeschiedenis.

- 1. Bent u ooit zwanger geweest
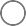
 Ja


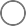
 Ja, op het moment zwanger
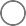
 Nooit zwanger geweest

#### If 'Bent u ooit zwanger geweest' is equal to 'Nooit zwanger geweest' answer this question:

De reden dat u nooit zwanger bent geweest heeft


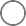
 niet te maken met de vasculaire malformatie
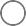
 wel te maken met de vasculaire malformatie

#### If 'De reden dat u nooit zwanger bent geweest heeft' is equal to 'wel te maken met de vasculaire malformatie' answer this question:

Kunt u toelichten waarom dit te maken had met de vasculaire malformatie?

#### If 'Bent u ooit zwanger geweest' is not equal to 'Nooit zwanger geweest' answer this question:

Heeft het hebben van een vasculaire malformatie

(aangeboren vaatafwijking) invloed gehad op uw keuze om wel of niet zwanger te worden?


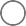
 Ja
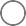
 Nee

#### If 'Heeft het hebben van een vasculaire malformatie (aangeboren vaatafwijking) invloed gehad op uw keuze om wel of niet zwanger te worden?' is equal to 'Ja'

***answer this question:***

Kunt u toelichten hoe het hebben van een vasculaire malformatie uw reproductieve keuzes heeft beïnvloed?

#### If 'Bent u ooit zwanger geweest' is not equal to 'Nooit zwanger geweest' answer this question:

Hoe vaak bent u zwanger geweest? Dit is inclusief een eventuele huidige zwangerschap en inclusief zwangerschappen die NIET hebben geleid tot de geboorte van een levend kind.


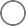
 1


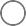
 2


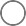
 3


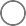
 4


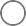
 5


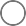
 6


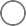
 7


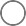
 8


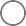
 9


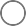
 Anders

#### If 'Hoe vaak bent u zwanger geweest? Dit is inclusief een eventuele huidige zwangerschap en inclusief zwangerschappen die NIET hebben geleid tot de geboorte van een levend kind.' is equal to '1' answer this question:

Is uw zwangerschap afgebroken (abortus)?


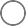
 Ja
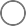
 Nee

#### If 'Is uw zwangerschap afgebroken (abortus)?' is equal to 'Ja' answer this question:

Had de reden voor de zwangerschapsafbreking (abortus) te maken met de vasculaire malformatie?


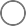
 Ja
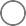
 Nee

#### If 'Had de reden voor de zwangerschapsafbreking (abortus) te maken met de vasculaire malformatie?' is equal to 'Ja' answer this question:

Wat is de reden dat de zwangerschapsafbreking (abortus) te maken had met de vasculaire malformatie?

#### If 'Hoe vaak bent u zwanger geweest? Dit is inclusief een eventuele huidige zwangerschap en inclusief zwangerschappen die NIET hebben geleid tot de geboorte van een levend kind.' is equal to '1' answer this question:

Heeft u een miskraam gehad? Een miskraam is een zwangerschap die spontaan en ongewild eindigt voor een zwangerschapsduur van 16 weken.


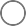
 Ja
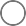
 Nee

#### If 'Hoe vaak bent u zwanger geweest? Dit is inclusief een eventuele huidige zwangerschap en inclusief zwangerschappen die NIET hebben geleid tot de geboorte van een levend kind.' is equal to '1' answer this question:

Heeft u een intra-uteriene vruchtdood gehad? Dit betekent het overlijden van een kind na 16 weken zwangerschapsduur en voor de geboorte.


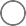
 Ja
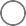
 Nee

#### If 'Hoe vaak bent u zwanger geweest? Dit is inclusief een eventuele huidige zwangerschap en inclusief zwangerschappen die NIET hebben geleid tot de geboorte van een levend kind.' is equal to '1' answer this question:

Heeft u een buitenbaarmoederlijke zwangerschap gehad?


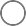
 Ja
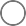
 Nee

#### If 'Hoe vaak bent u zwanger geweest? Dit is inclusief een eventuele huidige zwangerschap en inclusief zwangerschappen die NIET hebben geleid tot de geboorte van een levend kind.' is equal to '1' answer this question:

Bent u bevallen na een normale zwangerschapsduur? Dat wil zeggen na minstens 37 weken.


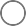
 Ja
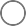
 Nee

#### If 'Hoe vaak bent u zwanger geweest? Dit is inclusief een eventuele huidige zwangerschap en inclusief zwangerschappen die NIET hebben geleid tot de geboorte van een levend kind.' is equal to '1' answer this question:

Heeft u een vroegtijdige bevallingen van een levend kind gehad?


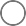
 Ja
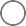
 Nee

U heeft aangegeven twee of meer keren zwanger te zijn geweest. Voor de volgende vragen wordt er soms om een toelichting gevraagd om welke zwangerschap het gaat. U dient dan te antwoorden met het 'nummer' van de zwangerschap. Uw eerste zwangerschap wordt '1', uw tweede zwangerschap '2', etc. Indien het meerdere zwangerschappen betreft scheidt u de getallen d.m.v. komma's (bij de eerste en tweede zwangerschap wordt: '1, 2').

#### If 'Hoe vaak bent u zwanger geweest? Dit is inclusief een eventuele huidige zwangerschap en inclusief zwangerschappen die NIET hebben geleid tot de geboorte van een levend kind.' is not equal to '1' answer this question:

Is er ooit een zwangerschap bij u afgebroken (abortus)?


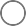
 Ja
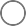
 Nee

- - - - 1. ***If 'Is er ooit een zwangerschap bij u afgebroken (abortus)?' is equal to 'Ja' answer this question:*** Hoe vaak is er een zwangerschap bij u afgebroken (abortus)? Antwoorden in een rond getal.

#### If 'Is er ooit een zwangerschap bij u afgebroken (abortus)?' is equal to 'Ja' answer this question:

Had de reden voor de zwangerschapsafbreking(en) (abortus) te maken met de vasculaire malformatie?


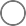
 Ja
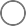
 Nee

#### If 'Had de reden voor de zwangerschapsafbreking(en) (abortus) te maken met de vasculaire malformatie?' is equal to 'Ja' answer this question:

Kunt u de reden hiervoor toelichten?

#### If 'Hoe vaak bent u zwanger geweest? Dit is inclusief een eventuele huidige zwangerschap en inclusief zwangerschappen die NIET hebben geleid tot de geboorte van een levend kind.' is not equal to '1' answer this question:

Heeft u ooit een miskraam gehad? Een miskraam is een zwangerschap die spontaan en ongewild eindigt voor een zwangerschapsduur van 16 weken.


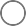
 Ja
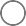
 Nee

#### If 'Heeft u ooit een miskraam gehad? Een miskraam is een zwangerschap die spontaan en ongewild eindigt

***voor een zwangerschapsduur van 16 weken.' is equal to 'Ja' answer this question:***

Hoe vaak heeft u een miskraam gehad? Antwoorden in een rond getal.

#### If 'Hoe vaak bent u zwanger geweest? Dit is inclusief een eventuele huidige zwangerschap en inclusief zwangerschappen die NIET hebben geleid tot de geboorte van een levend kind.' is not equal to '1' answer this question:

Heeft u ooit een intra-uteriene vruchtdood gehad? Dit betekent het overlijden van een kind na 16 weken zwangerschapsduur en voor de geboorte.


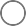
 Ja
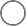
 Nee

#### If 'Heeft u ooit een intra-uteriene vruchtdood gehad? Dit betekent het overlijden van een kind na 16 weken

***zwangerschapsduur en voor de geboorte.' is equal to 'Ja' answer this question:***

Hoe vaak heeft u een intra-uteriene vruchtdood gehad? Antwoorden in een rond getal.

#### If 'Hoe vaak bent u zwanger geweest? Dit is inclusief een eventuele huidige zwangerschap en inclusief zwangerschappen die NIET hebben geleid tot de geboorte van een levend kind.' is not equal to '1' answer this question:

Heeft u ooit een buitenbaarmoederlijke zwangerschap gehad?


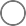
 Ja
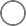
 Nee

#### If 'Heeft u ooit een buitenbaarmoederlijke zwangerschap gehad?' is equal to 'Ja' answer this question:

Hoe vaak heeft u een buitenbaarmoederlijke zwangerschap

gehad? Antwoorden in een rond getal.

#### If 'Hoe vaak bent u zwanger geweest? Dit is inclusief een eventuele huidige zwangerschap en inclusief zwangerschappen die NIET hebben geleid tot de geboorte van een levend kind.' is not equal to '1' answer this question:

Hoe vaak bent u bevallen na een normale zwangerschapsduur (dat wil zeggen minstens 37 weken) van een levend kind? Antwoorden in een rond getal.

# Vasculaire malformaties en zwangerschap vragenlijst - Medische voorgeschiedenis

### Number Question Answers

U heeft aangegeven nooit zwanger te zijn geweest. Daarom kunt u verder gaan naar het einde van de vragenlijst. De volgende vragen gaan over uw medische voorgeschiedenis.

#### If 'Bent u ooit zwanger geweest' is not equal to 'Nooit zwanger geweest' answer this question:

Heeft u, naast de vasculaire malformatie, een andere chronische ziekte?


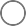
 Ja
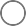
 Nee

- - 1. ***If 'Heeft u, naast de vasculaire malformatie, een andere chronische ziekte?' is equal to 'Ja' answer this question:*** Welke chronische ziekte heeft u?

#### If 'Bent u ooit zwanger geweest' is not equal to 'Nooit zwanger geweest' answer this question:

Heeft u ooit één of allebei deze aandoeningen gehad: trombose (bloedstolsel in bloedvat) of longembolie

(bloedstolsel in bloedvat longen)?

Ja Nee

# Vasculaire malformaties en zwangerschap vragenlijst - De periode vóór (of tussen) de zwangerschap(pen)

### Number Question Answers

U heeft aangegeven nooit trombose (bloedstolsel in bloedvat) of een longembolie (bloedstolsel in bloedvat longen) te hebben gehad. Daarom kunt via 'volgende' doorklikken naar de sectie over antistolling.

De volgende vragen gaan over de periode vóórdat u zwanger werd, of de periode tussen de zwangerschappen door. De vragen over de periode tijdens de zwangerschap en de kraamperiode van 6 weken na de zwangerschap komen later aan bod.

#### If 'Heeft u ooit één of allebei deze aandoeningen gehad: trombose (bloedstolsel in bloedvat) of longembolie (bloedstolsel in bloedvat longen)?' is equal to 'Ja' answer this question:

Heeft u vóór (of tussen) de zwangerschap(pen) **trombose** (bloedstolsel in bloedvat) gehad in een been, arm of in de buik?

Ja Nee

- - 1. ***If 'Heeft u vóór (of tussen) de zwangerschap(pen) trombose (bloedstolsel in bloedvat) gehad in een been, arm of in de buik?' is equal to 'Ja' answer this question:*** Wanneer heeft u vóór (of tussen) de zwangerschap(pen)

trombose gehad? Bijvoorbeeld voor de eerste zwangerschap, of tussen de tweede en de derde zwangerschap.

- - 1. ***If 'Heeft u vóór (of tussen) de zwangerschap(pen) trombose (bloedstolsel in bloedvat) gehad in een been, arm of in de buik?' is equal to 'Ja' answer this question:*** Waar had u precies trombose? Bijvoorbeeld 'linker onderbeen'.

#### If 'Heeft u ooit één of allebei deze aandoeningen gehad: trombose (bloedstolsel in bloedvat) of longembolie (bloedstolsel in bloedvat longen)?' is equal to 'Ja' answer this question:

Heeft u vóór (of tussen) de zwangerschap(pen) een **longembolie** gehad (bloedstolsel in bloedvat in de longen)?

Ja Nee

#### If 'Heeft u vóór (of tussen) de zwangerschap(pen) een longembolie gehad (bloedstolsel in bloedvat in de

***longen)?' is equal to 'Ja' answer this question:***

Wanneer heeft u vóór (of tussen) de zwangerschap(pen) een longembolie (bloedstolsel in bloedvat in de longen) gehad? Bijvoorbeeld voor de eerste zwangerschap, of tussen de tweede en de derde zwangerschap.

# Vasculaire malformaties en zwangerschap vragenlijst - Periode tijdens de zwangerschap

### Number Question Answers

U heeft aangegeven nooit trombose (bloedstolsel in bloedvat) of een longembolie (bloedstolsel in bloedvat longen) te hebben gehad. Daarom kunt via 'volgende' doorklikken naar de sectie over antistolling.

De volgende vragen gaan over de periode TIJDENS de zwangerschap. Vragen over de periode rondom de bevalling en de kraamperiode van 6 weken na de bevalling komen later aan bod.

#### If 'Heeft u ooit één of allebei deze aandoeningen gehad: trombose (bloedstolsel in bloedvat) of longembolie (bloedstolsel in bloedvat longen)?' is equal to 'Ja' answer this question:

Heeft u tijdens de zwangerschap(pen) **trombose**

(bloedstolsel in bloedvat) gehad in een been, arm of in de buik?

Ja Nee

#### If 'Heeft u tijdens de zwangerschap(pen) trombose (bloedstolsel in bloedvat) gehad in een been, arm of in de buik?' is equal to 'Ja' answer this question:

Tijdens welke zwangerschap(pen) heeft u trombose

(bloedstolsel in bloedvat) gehad? Antwoorden in ronde getallen; voor uw eerste zwangerschap vult u '1' in, voor uw tweede '2', etc.

#### If 'Heeft u tijdens de zwangerschap(pen) trombose (bloedstolsel in bloedvat) gehad in een been, arm of in de buik?' is equal to 'Ja' answer this question:

Waar had u precies trombose? Bijvoorbeeld 'linker onderbeen'.

#### If 'Heeft u ooit één of allebei deze aandoeningen gehad: trombose (bloedstolsel in bloedvat) of longembolie (bloedstolsel in bloedvat longen)?' is equal to 'Ja' answer this question:

Heeft u tijdens de zwangerschap een **longembolie** (bloedstolsel in bloedvat in de longen) gehad?

Ja Nee

#### If 'Heeft u tijdens de zwangerschap een longembolie (bloedstolsel in bloedvat in de longen) gehad?' is equal to 'Ja' answer this question:

Tijdens welke zwangerschap(pen) heeft u een longembolie

(bloedstolsel in bloedvat in de longen) gehad? Antwoorden in ronde getallen; voor uw eerste zwangerschap vult u '1' in, voor uw tweede '2', etc.

# Vasculaire malformaties en zwangerschap vragenlijst - Kraamperiode

### Number Question Answers

U heeft aangegeven nooit trombose (bloedstolsel in bloedvat) of een longembolie (bloedstolsel in bloedvat longen) te hebben gehad. Daarom kunt via 'volgende' doorklikken naar de sectie over antistolling.

De volgende vragen gaan over de kraamperiode, de periode van 6 weken na de bevalling(en).

#### If 'Heeft u ooit één of allebei deze aandoeningen gehad: trombose (bloedstolsel in bloedvat) of longembolie (bloedstolsel in bloedvat longen)?' is equal to 'Ja' answer this question:

Heeft u in de kraamperiode van 6 weken na de bevalling(en) **trombose** (bloedstolsel in bloedvat) gehad in een been, arm of in de buik?

Ja Nee

#### If 'Heeft u in de kraamperiode van 6 weken na de bevalling(en) trombose (bloedstolsel in bloedvat) gehad in een been, arm of in de buik?' is equal to 'Ja' answer this question:

Bij welke zwangerschap(pen) heeft u trombose (bloedstolsel in bloedvat) gehad in de kraamperiode van 6 weken na de bevalling? Antwoorden in ronde getallen; voor uw eerste zwangerschap vult u '1' in, voor uw tweede '2', etc.

#### If 'Heeft u in de kraamperiode van 6 weken na de bevalling(en) trombose (bloedstolsel in bloedvat) gehad in een been, arm of in de buik?' is equal to 'Ja' answer this question:

Waar had u precies trombose? Bijvoorbeeld 'linker onderbeen'.

#### If 'Heeft u ooit één of allebei deze aandoeningen gehad: trombose (bloedstolsel in bloedvat) of longembolie (bloedstolsel in bloedvat longen)?' is equal to 'Ja' answer this question:

Heeft u in de kraamperiode van 6 weken na de bevalling(en) een longembolie (bloedstolsel in bloedvat in de longen) gehad?

Ja Nee

#### If 'Heeft u in de kraamperiode van 6 weken na de bevalling(en) een longembolie (bloedstolsel in bloedvat in de longen) gehad?' is equal to 'Ja' answer this question:

Bij welke zwangerschap(pen) heeft u een longembolie gehad in de kraamperiode van 6 weken na de bevalling?

Antwoorden in ronde getallen; voor uw eerste zwangerschap vult u '1' in, voor uw tweede '2' etc.

# Vasculaire malformaties en zwangerschap vragenlijst - Antistolling

### Number Question Answers

De volgende vragen gaan over het gebruik van antistolling (bloedverdunners) rondom de zwangerschap.

#### If 'Bent u ooit zwanger geweest' is not equal to 'Nooit zwanger geweest' answer this question:

Bent u vóór (of tussen) de zwangerschap(pen) behandeld met antistollingsmedicijnen (bloedverdunners)?

Ja Nee

#### If 'Bent u vóór (of tussen) de zwangerschap(pen) behandeld met antistollingsmedicijnen

***(bloedverdunners)?' is equal to 'Ja' answer this question:*** Om welke redenen bent u behandeld met antistollingsmedicijnen (bloedverdunners)?

#### If 'Bent u vóór (of tussen) de zwangerschap(pen) behandeld met antistollingsmedicijnen

***(bloedverdunners)?' is equal to 'Ja' answer this question:*** Wanneer bent u vóór (of tussen) de zwangerschap(pen) behandeld met antistollingsmedicijnen (bloedverdunners) en voor hoe lang? Bijvoorbeeld 'na een trombosebeen 6 weken lang, vóór de eerste zwangerschap.' Als u dit niet meer weet vult u 'weet ik niet' in.

#### If 'Bent u vóór (of tussen) de zwangerschap(pen) behandeld met antistollingsmedicijnen

***(bloedverdunners)?' is equal to 'Ja' answer this question:***

Wat voor antistollingsmedicijnen (bloedverdunnende middelen) heeft u gebruikt? Als u dit niet meer weet vult u 'weet ik niet' in.

#### If 'Bent u ooit zwanger geweest' is not equal to 'Nooit zwanger geweest' answer this question:

Heeft u in de periode TIJDENS de zwangerschap(pen) of in de periode van 6 weken na de bevalling antistollingsmedicijnen (bloedverdunnende middelen) gebruikt?

Ja Nee

#### If 'Heeft u in de periode TIJDENS de zwangerschap(pen) of in de periode van 6 weken na de bevalling antistollingsmedicijnen (bloedverdunnende middelen)

***gebruikt?' is equal to 'Ja' answer this question:*** Om welke reden bent u behandeld met antistollingsmedicijnen (bloedverdunnende middelen)?

#### If 'Heeft u in de periode TIJDENS de zwangerschap(pen) of in de periode van 6 weken na de bevalling antistollingsmedicijnen (bloedverdunnende middelen)

***gebruikt?' is equal to 'Ja' answer this question:***

Bij welke zwangerschap(pen) heeft u antistollingsmedicijnen (bloedverdunnende middelen) gebruikt? Antwoorden in ronde getallen; voor uw eerste zwangerschap vult u '1' in, voor uw tweede '2', etc.

#### If 'Heeft u in de periode TIJDENS de zwangerschap(pen) of in de periode van 6 weken na de bevalling antistollingsmedicijnen (bloedverdunnende middelen)

***gebruikt?' is equal to 'Ja' answer this question:***

Kunt u toelichten wanneer in/na de zwangerschap(pen) u antistollingsmedicijnen heeft gebruikt en voor hoe lang? Bijvoorbeeld: vanaf de 10e week tot de 36e week zwangerschap, of vanaf de bevalling tot 6 weken erna. Als u dit niet meer weet vult u 'weet ik niet' in.

#### If 'Heeft u in de periode TIJDENS de zwangerschap(pen) of in de periode van 6 weken na de bevalling antistollingsmedicijnen (bloedverdunnende middelen)

***gebruikt?' is equal to 'Ja' answer this question:***

Wat voor antistollingsmedicijnen (bloedverdunnende middelen) heeft u gebruikt? Als u dit niet meer weet vult u 'weet ik niet' in.

# Vasculaire malformaties en zwangerschap vragenlijst - Klachten van de vasculaire malformatie tijdens de zwangerschap

### Number Question Answers

De volgende vragen gaan over de invloed van de zwangerschap op de klachten/symptomen die u ervaart ten gevolge van de vasculaire malformatie.

#### If 'Bent u ooit zwanger geweest' is not equal to 'Nooit zwanger geweest' answer this question:

Zijn de klachten die u heeft/had van de vasculaire malformatie toegenomen tijdens de zwangerschap(pen)?

Ja Nee

#### If 'Zijn de klachten die u heeft/had van de vasculaire malformatie toegenomen tijdens de zwangerschap(pen)?' is equal to 'Ja' answer this question:

Welke klachten zijn tijdens de zwangerschap toegenomen? Vink alle toepasselijke opties aan.

Pijn

Bloedingen

Bewegingsbeperking

Klachten van verstoord uiterlijk Ademhalingsproblemen

Lekkage van vocht Geen klachten

Anders

#### If 'Zijn de klachten die u heeft/had van de vasculaire malformatie toegenomen tijdens de zwangerschap(pen)?' is equal to 'Ja' answer this question:

Licht uw antwoord hier eventueel toe:

#### If 'Bent u ooit zwanger geweest' is not equal to 'Nooit zwanger geweest' answer this question:

Is de vasculaire malformatie tijdens de zwangerschap(pen) toegenomen in omvang/volume?

Ja Nee

# Vasculaire malformaties en zwangerschap vragenlijst - Complicaties tijdens de zwangerschap

### Number Question Answers

De volgende vragen gaan over complicaties tijdens de zwangerschap.

#### If 'Bent u ooit zwanger geweest' is not equal to 'Nooit zwanger geweest' answer this question:

Heeft u tijdens de zwangerschap(pen) andere complicaties gehad of bent u opgenomen geweest tijdens de zwangerschap?

Ja Nee

#### If 'Heeft u tijdens de zwangerschap(pen) andere complicaties gehad of bent u opgenomen geweest tijdens de zwangerschap?' is equal to 'Ja' answer this question:

Tijdens welke zwangerschap(pen) heeft u andere complicaties gehad of bent u opgenomen geweest? Antwoorden in ronde getallen; voor uw eerste zwangerschap vult u '1' in, voor uw tweede '2', etc.

#### If 'Heeft u tijdens de zwangerschap(pen) andere complicaties gehad of bent u opgenomen geweest tijdens de zwangerschap?' is equal to 'Ja' answer this question:

Kunt u de complicatie(s) beschrijven of de reden van opname geven?

# Vasculaire malformaties en zwangerschap vragenlijst - Complicaties rondom de bevalling en in de kraamperiode (6 weken na de bevalling)

### Number Question Answers

De volgende vragen gaan over de periode rondom de bevalling en de kraamperiode van 6 weken na de bevalling.

#### If 'Bent u ooit zwanger geweest' is not equal to 'Nooit zwanger geweest' answer this question:

Heeft u een keizersnede gehad?

Ja Nee

#### If 'Heeft u een keizersnede gehad?' is equal to 'Ja' answer this question:

Heeft u een **geplande** keizersnede gehad?

Ja Nee

#### If 'Heeft u een keizersnede gehad?' is equal to 'Ja' answer this question:

Bij welke zwangerschap(pen) heeft u een keizersnede gehad? Antwoorden in ronde getallen; voor uw eerste zwangerschap vult u '1' in, voor uw tweede '2', etc.

#### If 'Heeft u een keizersnede gehad?' is equal to 'Ja' answer this question:

Kunt u de reden voor de keizersnede toelichten?

#### If 'Bent u ooit zwanger geweest' is not equal to 'Nooit zwanger geweest' answer this question:

Had u voor/tijdens de vaginale bevalling of keizersnede een serieuze wens om een ruggenprik (epiduraal) te krijgen?

Ja Nee

#### If 'Had u voor/tijdens de vaginale bevalling of keizersnede een serieuze wens om een ruggenprik (epiduraal) te krijgen?' is equal to 'Ja' answer this question:

Heeft u tijdens de vaginale bevalling of keizersnede een ruggenprik (epiduraal) gehad?

Ja Nee

#### If 'Heeft u tijdens de vaginale bevalling of keizersnede een ruggenprik (epiduraal) gehad?' is equal to 'Nee'

***answer this question:***

Waarom heeft u geen ruggenprik (epiduraal) gekregen?

#### If 'Heeft u tijdens de vaginale bevalling of keizersnede een ruggenprik (epiduraal) gehad?' is equal to 'Ja'

***answer this question:***

Bij welke zwangerschap(pen) heeft u een ruggenprik (epiduraal) gekregen? Antwoorden in ronde getallen; voor uw eerste zwangerschap vult u '1' in, voor uw tweede '2', etc.

#### If 'Heeft u tijdens de vaginale bevalling of keizersnede een ruggenprik (epiduraal) gehad?' is equal to 'Ja'

***answer this question:***

Is er bij de ruggenprik(ken) (epiduraal) een bloeding ontstaan?

Ja Nee

#### If 'Bent u ooit zwanger geweest' is not equal to 'Nooit zwanger geweest' answer this question:

Heeft u tijdens de bevalling of in het kraambed meer dan 1 liter bloedverlies (fluxus) gehad?

Nee Ja

Weet ik niet

#### If 'Heeft u tijdens de bevalling of in het kraambed meer dan 1 liter bloedverlies (fluxus) gehad?' is equal to 'Ja' answer this question:

Tijdens welke zwangerschap(pen) heeft u meer dan 1 liter bloedverlies (fluxus) gehad? Antwoorden in ronde getallen; voor uw eerste zwangerschap vult u '1' in, voor uw tweede '2', etc.

#### If 'Bent u ooit zwanger geweest' is not equal to 'Nooit zwanger geweest' answer this question:

Heeft u tijdens de bevalling of in het kraambed een opname, een bloedtransfusie, of een operatie nodig gehad in verband met veel bloedverlies?

Ja Nee

#### If 'Heeft u tijdens de bevalling of in het kraambed een opname, een bloedtransfusie, of een operatie nodig gehad in verband met veel bloedverlies?' is equal to 'Ja' answer this question:

Tijdens welke zwangerschappen bent u opgenomen (gebleven) of behandeld in verband met veel bloedverlies?

#### If 'Heeft u tijdens de bevalling of in het kraambed een opname, een bloedtransfusie, of een operatie nodig gehad in verband met veel bloedverlies?' is equal to 'Ja' answer this question:

In verband met bloedverlies heb ik nodig gehad:

Opname in het ziekenhuis Bloedtransfusie

Operatie Anders

#### If 'Heeft u tijdens de bevalling of in het kraambed een opname, een bloedtransfusie, of een operatie nodig gehad in verband met veel bloedverlies?' is equal to 'Ja' answer this question:

Eventuele toelichting:

#### If 'Bent u ooit zwanger geweest' is not equal to 'Nooit zwanger geweest' answer this question:

Heeft u tijdens de bevalling(en) andere complicaties gehad?

Dit betekent dus NIET in de kraamperiode (6 weken na de zwangerschap).

Ja Nee

#### If 'Heeft u tijdens de bevalling(en) andere complicaties gehad? Dit betekent dus NIET in de kraamperiode (6 weken na de zwangerschap).' is equal to 'Ja' answer this question:

Bij welke bevalling(en) heeft u andere complicaties gehad? Antwoorden in ronde getallen; voor uw eerste zwangerschap vult u '1' in, voor uw tweede '2', etc.

#### If 'Heeft u tijdens de bevalling(en) andere complicaties gehad? Dit betekent dus NIET in de kraamperiode (6 weken na de zwangerschap).' is equal to 'Ja' answer this question:

Kunt u deze complicatie(s) beschrijven?

#### If 'Bent u ooit zwanger geweest' is not equal to 'Nooit zwanger geweest' answer this question:

Heeft u in de kraamperiode van 6 weken na de bevalling(en) andere complicaties gehad of bent u opgenomen geweest in het ziekenhuis?

Ja Nee

#### If 'Heeft u in de kraamperiode van 6 weken na de bevalling(en) andere complicaties gehad of bent u opgenomen geweest in het ziekenhuis?' is equal to 'Ja' answer this question:

Bij welke zwangerschap(pen) heeft u andere complicaties gehad of bent u opgenomen geweest in de kraamperiode van 6 weken na de bevalling?

#### If 'Heeft u in de kraamperiode van 6 weken na de bevalling(en) andere complicaties gehad of bent u opgenomen geweest in het ziekenhuis?' is equal to 'Ja' answer this question:

Kunt u een beschrijving geven van deze complicatie(s) of reden van opname?

## Onderzoek vasculaire malformaties en zwangerschap - Outro

Dit is het einde van de vragenlijst. Wij danken u hartelijk voor uw medewerking. Uw bijdrage zal ons helpen bij het verbeteren van ons begrip van deze aandoening en het mogelijk maken van een betere begeleiding van patiënten met vasculaire malformaties tijdens de zwangerschap.
