# Supplementary material for: Complications of Pregnancy and Birth in Women With Vascular Malformations: A Nationwide Cross‐Sectional Study
Source: BJOG. 2026 Mar 7;133(7):1493–501. doi: 10.1111/1471-0528.70205 (PMC13143545; doi:10.1111/1471-0528.70205)
Supplement: Supplementary file 2 — Data S2: bjo70205‐sup‐0002‐Supinfo2.docx. [file BJO-133-1493-s001.docx]

Survey Package 'Research on vascular malformations and pregnancy'

## Research on vascular malformations and pregnancy - Introduction

At the Department of Plastic Surgery at the Amsterdam University Medical Center (AUMC), we are conducting a new study into vascular malformations (congenital vascular abnormalities). At present, there is little clarity on how female patients with vascular malformations should be advised and treated when they want to become pregnant or are already pregnant.

For this reason, we are investigating the entire Dutch female patient population to determine whether there have been any difficulties with becoming pregnant, and whether any problems occurred during or shortly after pregnancy. This will allow us to provide better advice and potentially better support for patients with vascular malformations during pregnancy in the future.

You are now invited to complete a one-time questionnaire. The time it takes to complete the questionnaire depends on the number of pregnancies you have had. This can range from a few minutes (if you have never been pregnant) up to a maximum of 20 minutes. Even if you have never been pregnant, we kindly ask you to complete this questionnaire so that we can obtain as complete a picture as possible of the female patient population with vascular malformations. Please note that if you close or navigate away from the questionnaire before finishing, you will not be able to reopen it and continue where you left off. Therefore, ensure that you have sufficient time to complete the questionnaire in one sitting.

Your participation will help us gain a better understanding of this condition and contribute to improved guidance and care for women with vascular malformations.

Survey 'Vascular malformations and pregnancy questionnaire'

# Vascular malformations and pregnancy questionnaire - Personal data

### Number Question Answers

This questionnaire is intended for women aged 15 years and older with a congenital vascular defect (vascular malformation). If you do not fall in this category, you do not need to complete the questionnaire.

- 1. Though which route did you receive the invitation to participate in this study? (Please note: if you have been approached via both HEVAS and the Amsterdam UMC, you only need to complete the questionnaire once.)


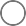
 E-mail from Amsterdam UMC
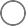
 E-mail from HEVAS


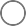
 Private Facebook group from HEVAS

- 1. Do you consent to the use of your personal data for this scientific study? The data will be processed anonymously in the study.


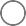
 Yes

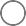
 No

Your data will be processed anonymously.

#### 1.2.2 If 'Do you consent to the use of your personal data for this scientific study? The data will be processed anonymously in the study.' is equal to 'Ja' answer this question:

Date of birth

*(dd-mm-yyyy)*

- 1. What is your current age? Years

#### If 'Do you consent to the use of your personal data for this scientific study? The data will be processed anonymously in the study.' is equal to 'Ja' answer this question:

Name (first and last name)

#### If 'Do you consent to the use of your personal data for this scientific study? The data will be processed anonymously in the study.' is equal to 'Ja' answer this question:

Do you give permission for medical data to be requested from your practitioner in the context of scientific research into vascular malformations? This data will be processed anonymously.


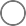
 Yes
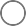
 No

# Vascular malformations and pregnancy questionnaire - Type of vascular malformation (congenital vascular anomaly)

### Number Question Answers

The following are a number of questions about the type of vascular malformation and the symptoms you experience.

- 1. In which hospital are you treated for the vascular malformation (congenital vascular anomaly)?
  2. What type of vascular malformation do you have? If you have a combined type, you can tick several types.


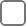
 Capillary malformation (port-wine stain)


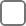
 Venous malformation (varicose veins/congenital defect of the veins)


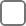
 Lymphatic malformation (abnormality of the lymphatic vessels)


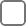
 Arteriovenous malformation (abnormality of the arteries)


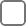
 Unclear

- 1. Where is the vascular malformation located?
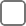
 Head/neck


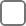
Chest/abdomen/pelvis
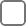
 Arms/hands

Legs/feet


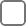


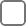
 Genital region
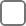
 Uterus

- 1. How big is the vascular malformation? (Measure or estimate the maximum diameter visible on the outside.)


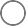
 Smaller than 5 cm

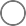
 5-10 cm


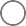
 10-20 cm


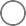
 20-30 cm


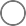
 Larger than 30 cm

- 1. Do you have body parts that are abnormal in size? This also includes, for example, leg lenght difference


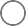
 Yes
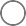
 No

#### If 'Do you have any body parts that are abnormal in size? This includes, for example,

***leg length difference.' is equal to 'yes' answer this question:***

Which body parts are different in size? Multiple answers possible. Check all applicable options.


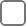
 Head/Neck
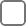
 Arms

Hands/fingers


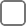


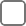
 Legs


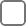
 Feet/toes


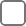
Chest/abdomen/pelvis/back
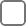
 Genital region

#### If 'Do you have any body parts that are abnormal in size? This includes, for example,

***leg length difference.' is equal to 'yes' answer this question:***

How does this body part differ in size/extent?

Longer


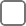


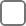
 Thicker
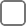
 Shorter
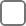
 Thinner
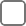
 Other

#### If 'Do you have any body parts that are abnormal in size/size? This includes, for example,

***leg length difference.' is equal to 'Ja' answer this question:***

If necessary, explanation:

- 1. Do you have the vascular malformation(s) as part of a syndrome? For example, Klippel-Trenaunay syndrome.


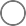
 Yes
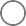
 No

#### If 'Do you have the vascular malformation(s) as part of a syndrome? For example, the Klippel-Trenaunay

***syndroom.' is equal to 'yes' answer this question:***

What syndrome do you have?

# Vascular malformations and pregnancy questionnaire - Symptoms of the vascular malformation and influence of hormones

### Number Question Answers

The following questions about the symptoms you experience from the vascular malformation and which factors have influenced this.

- 1. What symptoms do you experience from the vascular malformation? Multiple answers possible. Check all applicable options.


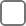
 Pain


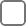
 Bleeding


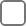
 Restriction of movement

Complaints of disturbed appearance


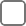


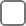
 Breathing problems


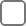
 Leakage of moisture
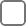
 No complaints


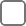
 Other

- 1. If necessary, explanation
  2. Did the symptoms of the vascular malformation start during puberty?


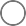
 Yes
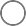
 No

- 1. Have the symptoms of the vascular malformation changed during puberty?


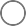
 Yes
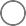
 No

- 1. If yes to the above questions: how did the symptoms of the vascular malformation start or change during puberty?
  2. Are the complaints/symptoms you experience as a result of your vascular malformation tied to your menstrual cycle?


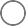
 Yes
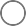
 No

- - 1. ***If 'Are the signs/symptoms you experience as a result of your vascular malformation tied to your menstrual cycle?' is equal to 'yes' answer this question:*** How do the signs/symptoms change during your cycle?
  1. Have you ever used hormonal contraception? For example, the contraceptive pill or a hormonal IUD.


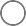
 Yes
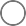
 No

#### If 'Have you ever used hormonal birth control? For example, the contraceptive pill or a hormonal IUD.' is equal to 'yes' answer this question:

Have the symptoms of the vascular malformation changed during the use of hormonal contraception?


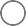
 Yes
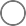
 No

#### If 'Did the symptoms of the vascular malformation change during the use of the hormonal contraception?' is equal to 'yes' answer this question: What changed in the symptoms during the use of hormonal contraception?

# Vascular malformations and pregnancy questionnaire - Obstetric history

### Number Question Answers

The following questions are about your obstetric history.

- 1. Have you ever been pregnant
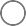
 Yes


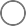
 Yes, I am currently pregnant
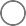
 I have never been pregnant

#### If 'Have you ever been pregnant' is equal to 'I have never been pregnant' answer this question:

The reason you have never been pregnant

Is related to the vascular malformation


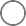


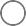
 Is not related to the vascular malformation

#### If 'The reason you have never been pregnant' is equal to 'Is related to the vascular malformation' answer this question:

Can you explain why this is related to the vascular malformation?

#### If 'Have you ever been pregnant' is not equal to 'Never been pregnant' answer this question:

Has having a vascular malformation

(congenital vascular defect) influenced your choice whether or not to become pregnant?


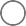
 Yes
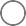
 No

#### If 'Did having a vascular malformation (congenital vascular abnormality) affect your choice whether or not to become pregnant?' is equal to 'Yes'

***answer this question:***

Can you elaborate on how having a vascular malformation has affected your reproductive choices?

#### If 'Have you ever been pregnant' is not equal to 'Never been pregnant' answer this question:

How many times have you been pregnant? This includes any current pregnancy and includes pregnancies that did NOT result in the birth of a live child.


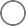
 1


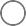
 2


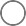
 3


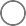
 4


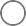
 5


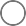
 6


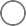
 7


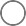
 8


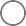
 9


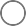
 Other

#### If 'How many times have you been pregnant? This includes any current pregnancy and includes pregnancies that did NOT result in the birth of a live child.' is equal to '1' answer this question:

Has your pregnancy been terminated (abortion)?


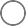
 Yes
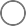
 No

#### If 'Has your pregnancy been terminated (abortion)?' is equal to 'yes' answer this question:

Did the reason for the termination of pregnancy (abortion) have to do with the vascular malformation?

Yes
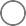
 No


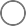


#### If 'Did the reason for the termination of pregnancy (abortion) have to do with the vascular malformation?' is equal to 'yes' answer this question:

Why did the termination of pregnancy (abortion) have to do with the vascular malformation?

#### If 'How many times have you been pregnant? This includes any current pregnancy and includes pregnancies that did NOT result in the birth of a live child.' is equal to '1' answer this question:

Have you had a miscarriage? A miscarriage is a pregnancy that ends spontaneously and unintentionally before a gestational age of 16 weeks.


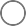
 Yes
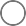
 No

#### If 'How many times have you been pregnant? This includes any current pregnancy and includes pregnancies that did NOT result in the birth of a live child.' is equal to '1' answer this question:

Have you had an intrauterine fetal death? This means the death of a child after 16 weeks of gestational age and before birth.


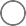
 Yes
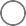
 No

#### If 'How many times have you been pregnant? This includes any current pregnancy and includes pregnancies that did NOT result in the birth of a live child.' is equal to '1' answer this question:

Have you had an ectopic pregnancy?


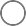
 Yes
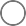
 No

#### If 'How many times have you been pregnant? This includes any current pregnancy and includes pregnancies that did NOT result in the birth of a live child.' is equal to '1' answer this question:

Did you give birth after a normal pregnancy duration? That means after at least 37 weeks.


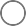
 Yes
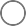
 No

#### If 'How many times have you been pregnant? This includes any current pregnancy and includes pregnancies that did NOT result in the birth of a live child.' is equal to '1' answer this question:

Have you had premature delivery of a live child?


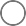
 Yes
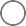
 No

You have indicated that you have been pregnant two or more times. For the following questions, an explanation is sometimes asked for which pregnancy it concerns. You must then answer with the 'number' of the pregnancy. Your first pregnancy will be '1', your second pregnancy '2', etc. If there are multiple pregnancies, separate the numbers by means of commas (for the first and second pregnancy becomes: '1, 2').

#### If 'How many times have you been pregnant? This includes any current pregnancy and includes pregnancies that did NOT result in the birth of a live child.' is not equal to '1' answer this question:

Have you ever had a pregnancy terminated (abortion)?

Yes
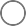
 No


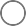


- - - - 1. ***If 'Have you ever had a pregnancy terminated (abortion)?' is equal to 'yes' answer this question:*** How often have you had a pregnancy terminated (abortion)? Answers in a round number.

#### If 'Have you ever had a pregnancy terminated (abortion)?' is equal to 'yes' answer this question:

Did the reason for the termination of pregnancy(s) (abortion) have to do with the vascular malformation?


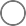
 Yes
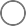
 No

#### If 'Did the reason for the termination of pregnancy(s) (abortion) have to do with the vascular malformation?' is equal to 'yes' answer this question:

Can you explain the reason for this?

#### If 'How many times have you been pregnant? This includes any current pregnancy and includes pregnancies that did NOT result in the birth of a live child.' is not equal to '1' answer this question:

Have you ever had a miscarriage? A miscarriage is a pregnancy that ends spontaneously and unintentionally before a gestational age of 16 weeks.


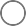
 Yes
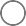
 No

#### If 'Have you ever had a miscarriage? A miscarriage is a pregnancy that ends spontaneously and unintentionally before a gestational age of 16 weeks.' is equal to 'Ja' answer this question:

How often have you had a miscarriage? Answer in a round number.

#### If 'How many times have you been pregnant? This includes any current pregnancy and includes pregnancies that did NOT result in the birth of a live child.' is not equal to '1' answer this question:

Have you ever had intrauterine fetal death? This means the death of a child after 16 weeks of gestational age and before birth.


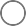
 Yes
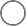
 No

#### If 'Have you ever had an intrauterine fetal death? This means the death of a child after 16 weeks

***gestational age and before birth.' is equal to 'yes' answer this question:***

How often have you had intrauterine fetal death? Answer in a round number.

#### If 'How many times have you been pregnant? This includes any current pregnancy and includes pregnancies that did NOT result in the birth of a live child.' is not equal to '1' answer this question:

Have you ever had an ectopic pregnancy?


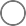
 Yes
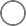
 No

#### If 'Have you ever had an ectopic pregnancy?' is equal to 'yes' answer this question:

How often do you have an ectopic pregnancy?

Answer in a round number.

#### If 'How many times have you been pregnant? This includes any current pregnancy and includes pregnancies that did NOT result in the birth of a live child.' is not equal to '1' answer this question:

How many times have you given birth to a live child after a normal gestational age (i.e. at least 37 weeks)? Answer in a round number.

# Vascular malformations and pregnancy questionnaire - Medical history

### Number Question Answers

You have indicated that you have never been pregnant. Therefore, you can proceed to the end of the questionnaire. The following questions are about your medical history.

#### If 'Have you ever been pregnant' is not equal to 'Never been pregnant' answer this question:

Do you have another chronic disease besides the vascular malformation?


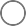
 Yes
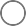
 No

- - 1. ***If 'Do you have another chronic disease besides the vascular malformation?' is equal to 'Yes' answer this question:*** Which chronic disease do you have?

#### If 'Have you ever been pregnant' is not equal to 'Never been pregnant' answer this question:

Have you ever had one or both of these conditions: thrombosis (blood clot in blood vessels) or pulmonary embolism (blood clot in blood vessels in the lungs)?

Yes No

# Vascular malformations and pregnancy questionnaire - The period before (or between) the pregnancy(s)

### Number Question Answers

You have indicated that you have never had thrombosis (blood clot in blood vessels) or pulmonary embolism (blood clot in blood vessels of the lungs). That is why you can click through to the section on anticoagulation via 'next'.

The following questions are about the period before you became pregnant, or the period between pregnancies. The questions about the period during pregnancy and the postpartum period of 6 weeks after pregnancy will be discussed later.

#### If 'Have you ever had one or both of these conditions: thrombosis (blood clot in blood vessels) or pulmonary embolism (blood clot in blood vessels of the lungs)?' is equal to 'yes' answer this question:

Have you had thrombosis (blood clot in blood vessels) in a leg, arm or abdomen before (or between) the pregnancy(s)?

Yes No

- - 1. ***If 'Have you had thrombosis (blood clot in blood vessels) in a leg, arm or abdomen before (or between) the pregnancy(ies)?' is equal to 'yes' answer this question:*** *When did you have a thrombosis (blood clot in the blood vessel)* before (or between) the pregnancy(s)

Had thrombosis? For example, before the first pregnancy, or between the second and third pregnancy.

- - 1. ***If 'Did you have thrombosis (blood clot in blood vessel) in a leg, arm or abdomen before (or between) the pregnancy(s)?' is equal to 'yes' answer this question:*** Where exactly did you have thrombosis? For example, 'left lower leg'.

#### If 'Have you ever had one or both of these conditions: thrombosis (blood clot in blood vessels) or pulmonary embolism (blood clot in blood vessels of the lungs)?' is equal to 'yes' answer this question:

Have you had a pulmonary embolism (blood clot in the blood vessels in the lungs) before (or between) pregnancy(s)?

Yes No

#### If 'Have you had a pulmonary embolism before (or between) pregnancy(s) (blood clot in blood vessels in the

***lungs)?' is equal to 'Ja' answer this question:***

When did you have a pulmonary embolism (blood clot in blood vessels in the lungs) before (or between) the pregnancy(s)? For example, before the first pregnancy, or between the second and third pregnancy.

# Vascular malformations and pregnancy questionnaire - Period during pregnancy

### Number Question Answers

You have indicated that you have never had thrombosis (blood clot in blood vessels) or pulmonary embolism (blood clot in blood vessels lungs). That is why you can click through to the section on anticoagulation via 'next'.

The following questions are about the period DURING pregnancy. Questions about the period around childbirth and the postpartum period of 6 weeks after delivery will be discussed later.

#### If 'Have you ever had one or both of these conditions: thrombosis (blood clot in blood vessels) or pulmonary embolism (blood clot in blood vessels of the lungs)?' is equal to 'yes' answer this question:

Did you have thrombosis (blood clot in blood vessel) in a leg, arm or abdomen **during pregnancy(s)**?

Yes No

#### If 'Have you had thrombosis (blood clot in blood vessels) in a leg, arm or abdomen during pregnancy(s)?' is equal to 'yes' answer this question:

During which pregnancy(s) did you have thrombosis

(blood clot in blood vessel)? Answers in round numbers; For your first pregnancy you enter '1', for your second '2', etc.

#### If 'Have you had thrombosis (blood clot in blood vessels) in a leg, arm or abdomen during pregnancy(s)?' is equal to 'yes' answer this question:

Where exactly did you have thrombosis? For example, 'left lower leg'.

#### If 'Have you ever had one or both of these conditions: thrombosis (blood clot in blood vessels) or pulmonary embolism (blood clot in blood vessels of the lungs)?' is equal to 'yes' answer this question:

Have you had a **pulmonary embolism** (blood clot in blood vessels in the lungs) during pregnancy?

Yes No

#### If 'Did you have a pulmonary embolism (blood clot in blood vessels in the lungs) during pregnancy?' is equal to 'yes' answer this question:

During which pregnancy(s) did you have a pulmonary embolism?

(blood clot in blood vessel in the lungs)? Answers in round numbers; For your first pregnancy you enter '1', for your second '2', etc.

# Vascular malformations and pregnancy questionnaire - Postpartum period

### Number Question Answers

You have indicated that you have never had thrombosis (blood clot in blood vessels) or pulmonary embolism (blood clot in blood vessels lungs). That is why you can click through to the section on anticoagulation via 'next'.

The next questions are about the postpartum period, the period of 6 weeks after the birth(s).

#### If 'Have you ever had one or both of these conditions: thrombosis (blood clot in blood vessels) or pulmonary embolism (blood clot in blood vessels lungs)?' is equal to 'yes' answer this question:

Have you had thrombosis (blood clot in blood vessel) in a leg, arm or abdomen during the postpartum period of 6 weeks after childbirth?

Yes No

#### If 'Have you had thrombosis (blood clot in blood vessel) in a leg, arm or abdomen in the postpartum period of 6 weeks after delivery?' is equal to 'yes' answer this question:

After which pregnancy(s) did you have thrombosis (blood clot in blood vessel) in the postpartum period of 6 weeks after childbirth? Answers in round numbers; For your first pregnancy you enter '1', for your second '2', etc.

#### If 'Have you had thrombosis (blood clot in blood vessel) in a leg, arm or abdomen in the postpartum period of 6 weeks after childbirth?' is equal to 'yes' answer this question:

Where exactly did you have thrombosis? For example, 'left lower leg'.

#### If 'Have you ever had one or both of these conditions: thrombosis (blood clot in blood vessels) or pulmonary embolism (blood clot in blood vessels lungs)?' is equal to 'yes' answer this question:

Have you had a pulmonary embolism (blood clot in blood vessels in the lungs) during the postpartum period of 6 weeks after childbirth?

Yes No

#### If 'Have you had a pulmonary embolism (blood clot in blood vessels in the lungs) during the postpartum period of 6 weeks after delivery(s)?' is equal to 'Yes' answer this question:

In which pregnancy(s) did you have a pulmonary embolism in the postpartum period of 6 weeks after delivery?

Answers in round numbers; For your first pregnancy you enter '1', for your second '2' etc.

# Vascular malformations and pregnancy questionnaire - Anticoagulation

### Number Question Answers

The following questions are about the use of anticoagulation (blood thinners) around pregnancy.

#### If 'Have you ever been pregnant' is not equal to 'Never been pregnant' answer this question:

Have you been treated with anticoagulant medication (blood thinners) before (or between) pregnancy(s)?

Yes No

#### If 'Have you been treated with anticoagulant medication before (or between) pregnancy(s)

***(blood thinners)?' is equal to 'yes' answer this question:*** For what reasons have you been treated with anticoagulant medication (blood thinners)?

#### If 'Have you been treated with anticoagulant medication before (or between) pregnancy(s)

***(blood thinners)?' is equal to 'yes' answer this question:*** When were you treated with anticoagulant medication (blood thinners) before (or between) the pregnancy(s) and for how long? For example, 'after a thrombosis in the leg for 6 weeks, before the first pregnancy.' If you don't remember this, fill in 'I don't know'.

#### If 'Have you been treated with anticoagulant medication before (or between) pregnancy(s)

***(blood thinners)?' is equal to 'yes' answer this question:***

What kind of anticoagulant medication (blood thinners) have you used? If you don't remember this, fill in 'I don't know'.

#### If 'Have you ever been pregnant' is not equal to 'Never been pregnant' answer this question:

Have you been treated with anticoagulant medication (blood thinners) in the period DURING pregnancy(s) or in the period of 6 weeks after childbirth?

Yes No

#### If ‘Have you been treated with anticoagulant medication (blood thinners) in the period DURING pregnancy(s) or in the period of 6 weeks after childbirth?’ Is equal to 'Yes' answer this question: For what reason have you been treated with anticoagulant medication (blood thinners)?

#### If 'Have you been treated with anticoagulant medication (blood thinners) in the period DURING pregnancy(s) or in the period of 6 weeks after childbirth?’ Is equal to 'yes' answer this question:

During which pregnancy(s) did you take anticoagulant medication (blood thinners)? Answers in round numbers; For your first pregnancy you enter '1', for your second '2', etc.

#### If 'Have you been treated with anticoagulant medication (blood thinners) in the period DURING pregnancy(s) or in the period of 6 weeks after childbirth?’ Is equal to 'yes' answer this question:

Can you explain when you used anticoagulant medication during or after the pregnancy(s) and for how long? For example: from the 10th week to the 36th week of pregnancy, or from delivery to 6 weeks after. If you don't remember this, fill in 'I don't know'.

#### If 'Have you been treated with anticoagulant medication (blood thinners) in the period DURING pregnancy(s) or in the period of 6 weeks after childbirth?

***is equal to 'yes' answer this question:***

What kind of anticoagulants (blood thinners) have you used? If you don't remember this, fill in 'I don't know'.

# Vascular malformations and pregnancy questionnaire - Symptoms of the vascular malformation during pregnancy

### Number Question Answers

The following questions are about the influence of pregnancy on the symptoms you experienced related to the vascular malformation.

#### If 'Have you ever been pregnant' is not equal to 'Never been pregnant' answer this question:

Did the symptoms you experience from the vascular malformation increase during the pregnancy(s)?

Yes No

#### If ‘Did the symptoms you experience from the vascular malformations increase during pregnancy(s)' is equal to 'yes' answer this question:

Which symptoms increased during pregnancy? Check all applicable options.

Pain

Bleeding

Restriction of movement

Complaints of disturbed appearance

Breathing problems

Leakage of moisture No complaints

Other

#### If ‘Did the symptoms you experience from the vascular malformation increase during pregnancy' is equal to 'yes' answer this question:

If necessary, please explain your answer here:

#### If 'Have you ever been pregnant' is not equal to 'Never been pregnant' answer this question:

Did the vascular malformation increase in size/volume during pregnancy(s)?

Yes No

# Vascular malformations and pregnancy questionnaire - Complications during pregnancy

### Number Question Answers

The following questions are about complications during pregnancy.

#### If 'Have you ever been pregnant' is not equal to 'Never been pregnant' answer this question:

Have you had other complications during the pregnancy(s) or have you been hospitalized during the pregnancy?

Yes No

#### If 'Have you had any other complications during your pregnancy(s) or have you been hospitalized during pregnancy?' is equal to 'yes' answer this question:

During which pregnancy(s) did you have other complications or were you hospitalized? Answers in round numbers; For your first pregnancy you enter '1', for your second '2', etc.

#### If 'Have you had any other complications during your pregnancy(s) or have you been hospitalized during pregnancy?' is equal to 'yes' answer this question:

Can you describe the complication(s) or give the reason for admission?

# Vascular malformations and pregnancy questionnaire - Complications around childbirth and in the postpartum period (6 weeks after delivery)

### Number Question Answers

The following questions are about the period around childbirth and the postpartum period of 6 weeks after childbirth.

#### If 'Have you ever been pregnant' is not equal to 'Never been pregnant' answer this question:

Have you had a caesarean section?

Yes No

#### If 'Have you had a caesarean section?' is equal to 'Ja' answer this question:

Have you had a **planned** caesarean section?

Yes No

#### If 'Have you had a caesarean section?' is equal to 'yes' answer this question:

During which pregnancy(s) did you have a caesarean section? Answers in round numbers; For your first pregnancy you enter '1', for your second '2', etc.

#### If 'Have you had a caesarean section?' is equal to 'yes' answer this question:

Can you explain the reason for the caesarean section?

#### If 'Have you ever been pregnant' is not equal to 'Never been pregnant' answer this question:

Did you have a serious/strong desire to get an epidural before/during the vaginal delivery or caesarean section?

Yes No

#### If 'Did you have a serious wish to get an epidural before/during the vaginal delivery or caesarean section?' is equal to 'Ja' answer this question:

Did you have an epidural during the vaginal delivery or caesarean section?

Yes No

#### If 'Did you have an epidural during the vaginal delivery or caesarean section?' is equal to 'No'

***answer this question:***

Why didn't you get an epidural?

#### If 'Did you have an epidural during the vaginal delivery or caesarean section?' is equal to 'Yes'

***answer this question:***

In which pregnancy(s) did you receive an epidural? Answers in round numbers; For your first pregnancy you enter '1', for your second '2', etc.

#### If 'Did you have an epidural during the vaginal delivery or caesarean section?' is equal to 'Yes'

***answer this question:***

Did the epidural(s) cause bleeding?

Yes No

#### If 'Have you ever been pregnant' is not equal to 'Never been pregnant' answer this question:

Did you experience more than 1 litre of blood loss (postpartum haemorrhage) during childbirth or in the postpartum period?

No Yes

I don't know

#### If 'Did you experience more than 1 litre of blood loss (postpartum haemorrhage) during childbirth or during childbirth?' is equal to 'yes' answer this question:

During which pregnancy(s) did you experience more than 1 liter of blood loss (postpartum haemorrhage)? Answer in round numbers; For your first pregnancy you enter '1', for your second '2', etc.

#### If 'Have you ever been pregnant' is not equal to 'Never been pregnant' answer this question:

Did you require (prolonged) hospitalization, a blood transfusion, or surgery during childbirth or in the postpartum period due to severe blood loss?

Yes No

#### If 'Did you require (prolonged) hospitalization, a blood transfusion, or surgery during childbirth or in the postpartum period due to severe blood loss?' is equal to 'yes' answer this question:

During which pregnancies have you been hospitalized or treated for severe blood loss?

#### If 'Did you require (prolonged) hospitalization, a blood transfusion, or surgery during childbirth or in the postpartum period due to severe blood loss' is equal to 'Ja' answer this question:

Because of severe blood loss I required the following:

(prolonged) hospitalization Blood transfusion

Surgery
 Other

#### If 'Did you require (prolonged) hospitalization, a blood transfusion, or surgery during childbirth or in the postpartum period due to severe blood loss?' is equal to 'yes' answer this question:

If needed, you can provide an explanation here:

#### If 'Have you ever been pregnant' is not equal to 'Never been pregnant' answer this question:

Did you experience any othere complications during labor or delivery? This does NOT include the postpartum period (6 weeks after childbirth).

Yes No

#### If 'Did you experience any other complications during labour or delivery? This does NOT include the postpartum period (6 weeks after pregnancy).' is equal to 'yes' answer this question:

During which deliveries did you experience other complications? Answer in round numbers; For your first pregnancy you enter '1', for your second '2', etc.

#### If 'Did you have any other complications during labour or delivery? This does NOT include the postpartum period (6 weeks after pregnancy).' is equal to 'yes' answer this question:

Can you describe these complication(s)?

#### If 'Have you ever been pregnant' is not equal to 'Never been pregnant' answer this question:

Did you experience any other complications or have you been admitted to the hospital during the 6 week postpartum period after your delivery or deliveries?

Yes No

#### If 'Did you experience any other complications or have you been admitted to the hospital during the 6 week postpartum period after your delivery or deliveries?' is equal to 'yes' answer this question:

During which pregnancy(s) have you had other complications or have you been admitted during the postpartum period of 6 weeks after delivery?

#### If ‘Did you experience any other complications or have you been admitted to the hospital during the 6 week postpartum period after your delivery or deliveries?' is equal to 'yes' answer this question:

Can you describe these complication(s) or reasons for hospital admission?

## Research on vascular malformations and pregnancy - Outro

This is the end of the questionnaire. We sincerely thank you for your cooperation. Your contribution will help us improve our understanding of this condition and enable better care for patients with vascular malformations during pregnancy.
