# Supplementary material for: Complications of Pregnancy and Birth in Women With Vascular Malformations: A Nationwide Cross‐Sectional Study
Source: BJOG. 2026 Mar 7;133(7):1493–501. doi: 10.1111/1471-0528.70205 (PMC13143545; doi:10.1111/1471-0528.70205)
Supplement: Supplementary file 3 — Table S1: Baseline characteristics of patients who had at least one birth, comparing patients recruited through Amsterdam University Medical Centre and HEVAS. [file BJO-133-1493-s003.docx]

**Supplementary material

Appendix S1.** PubMed search strategy for identifying population-based cohort studies and meta-analyses conducted within the last 15 years, focusing on complications of miscarriage, caesarean section, deep venous thrombosis, pulmonary embolism, and postpartum hemorrhage. **Venous Thromboembolism**

("Venous Thromboembolism"[Mesh] OR "Pulmonary Embolism"[Mesh] OR "Venous Thrombosis"[Mesh] OR "Deep Vein Thrombosis"[Mesh] OR "Thromboembolism"[Mesh] OR thromboembol*[tiab] OR embolism[tiab] OR "DVT"[tiab] OR "deep vein thrombosis"[tiab]

OR "venous thrombosis"[tiab]) AND ("Pregnancy"[Mesh] OR pregnan*[tiab]) AND (incidence[tiab] OR prevalence[tiab] OR population*[tiab])

**Postpartum Hemorrhage**

("Postpartum Hemorrhage"[Mesh] OR "postpartum hemorrhag*"[tiab] OR "postpartum haemorrhag*"[tiab] OR PPH[tiab]) AND (incidence[tiab] OR prevalence[tiab] OR rate*[tiab]) AND ("population-based"[tiab] OR cohort[tiab])

**Cesarean Section**

("Cesarean Section"[Mesh] OR "caesarean section*"[tiab] OR "c-section"[tiab]) AND

(incidence[tiab] OR prevalence[tiab] OR rate*[tiab] OR percentage[tiab] OR frequency[tiab])

AND ("population-based"[tiab] OR cohort[tiab] OR "national data"[tiab] OR "registry"[tiab])

**Miscarriage**

("Abortion, Spontaneous"[Mesh] OR "spontaneous abortion"[tiab] OR miscarriage[tiab]) AND

(incidence[tiab] OR prevalence[tiab] OR rate*[tiab] OR percentage[tiab] OR frequency[tiab]) AND

("population-based"[tiab] OR cohort[tiab] OR "registry"[tiab] OR "national data"[tiab])

**Table S1.** Baseline characteristics of patients who had at least one birth, comparing patients recruited through Amsterdam University Medical Centre and HEVAS.

|  | AUMC-recruited | HEVAS-recruited | p-value |
| --- | --- | --- | --- |
| Number of patients | 67 | 38 |  |
| Age in years when completing questionnaire, median (IQR) | 44 (37-58) | 48 (36-63) | 0.67 |
| Vascular malformations, n (%) |  |  |  |
| CM | 20 (29.9) | 15 (39.5) | 0.32 |
| VM | 42 (62.7) | 29 (76.3) | 0.15 |
| AVM | 5 (7.5) | 7 (18.4) | 0.09 |
| LM | 8 (11.9) | 5 (13.2) | 0.86 |
| Unclear | 14 (20.9) | 1 (2.6) | 0.01 |
| Anatomical location, n (%) |  |  |  |
| Lower extremities | 29 (43.3) | 27 (71.1) | 0.01 |
| Trunk | 14 (20.9) | 13 (34.2) | 0.13 |
| External genitalia | 16 (23.9) | 7 (18.4) | 0.52 |
| Uterus | 6 (9.0) | 5 (13.1) | 0.50 |
| Upper extremities | 13 (19.4) | 11 (22.9) | 0.26 |
| Head and neck | 25 (33.0) | 13 (34.2) | 0.13 |
| Malformation size, n (%) |  |  | 0.01 |
| < 5 cm | 17 (25.4) | 2 (5.3) |  |
| 5-10 cm | 21 (31.3) | 5 (13.2) |  |
| 10-20 cm | 8 (11.9) | 8 (21.1) |  |
| 20-30 cm | 2 (3.0) | 2 (5.3) |  |
| >30 cm | 19 (28.4) | 21 (55.3) |  |
| Tissue overgrowth, n (%) | 29 (43.3) | 26 (68.4) | 0.01 |
| Syndrome, n (%) | 15 (22.4) | 22 (57.9) | <0.01 |
| Klippel-trenaunay | 15 (27.3) | 19 (50) |  |
| Parkes Weber | 0 | 3 (7.9) |  |
| VM-related Symptoms, n (%)* |  |  |  |
| Pain | 44 (34.3) | 34 (89.5) | 0.01 |
| Impaired mobility | 12 (17.9) | 6 (15.8) | 0.71 |
| Disfigurement | 31 (46.3) | 20 (52.6) | 0.53 |
| Bleeding | 12 (17.9) | 6 (15.8) | 0.78 |
| Fluid leakage | 2 (3.0) | 2 (5.3) | 0.56 |
| Breathing issues | 5 (7.5) | 2 (5.3) | 0.66 |
| Asymptomatic | 4 (6.0) | 0 | 0.13 |
| Other symptoms | 13 (19.4) | 6 (15.8) | 0.64 |

AVM = arteriovenous malformation, CM = capillary malformation, IQR = interquartile range, LM = lymphatic malformation, VM = venous malformation.
*Pre-existing VM-related symptoms, independent of pregnancy.
